# SIK2-mediated phosphorylation of GABARAPL2 facilitates autophagosome–lysosome fusion and rescues neurodegeneration in an Alzheimer’s disease model

**DOI:** 10.1186/s40035-025-00514-4

**Published:** 2025-10-23

**Authors:** Xiaoman Dai, Ziling Ye, Chen Wang, Yufei Huang, Yun Chen, Tianqing Han, Weijie Gao, Xin Wu, Jing Zhang, Xiaochun Chen

**Affiliations:** 1 Department of Neurology and Geriatrics, Fujian Institute of Geriatrics, Fujian Medical University Union Hospital, Fuzhou, 350001 China; 2 Fujian Key Laboratory of Molecular Neurology and Institute of Neuroscience, School of Basic Medical Sciences, Fujian Medical University, Fuzhou, 350001 China

**Keywords:** Autophagic flux, Salt-inducible kinase 2, Alzheimer's disease, GABARAPL2, Autophagosome–lysosome fusion, Neurodegeneration

## Abstract

**Background:**

Defective autophagic flux is implicated in Alzheimer's disease (AD), but the molecular mechanisms underlying this process are not fully understood. Salt-inducible kinase 2 (SIK2) is associated with autophagic function. However, its specific involvement in autophagic flux regulation and AD pathogenesis remains unclear.

**Methods:**

We evaluated hippocampal SIK2 expression and its age-related changes in postmortem AD patients and 5 × FAD mice by bioinformatics analysis, immunofluorescence, qPCR, and Western blotting. To investigate the functional role of SIK2, we employed adeno-associated virus-mediated SIK2 knockdown and overexpression in combination with behavioral tests (Morris water maze), electrophysiological recordings (long-term potentiation, LTP), and ultrastructural analysis (electron microscopy) to evaluate cognitive function and synaptic plasticity. Autophagic flux was measured using LC3B/p62 turnover assays, mRFP-GFP-LC3 tandem fluorescence assay, and transmission electron microscopy. Mechanistic insights were gained through co-immunoprecipitation assay, GST-pull down assay, phosphoproteomics, and site-directed mutagenesis. Additionally, phosphorylation-mimetic (S72E) and non-phosphorylatable (S72A) mutants of GABA type A receptor-associated protein-like 2 (GABARAPL2) were intrahippocampally delivered to 5 × FAD mice to explore their effects.

**Results:**

Our study identified SIK2 as a critical regulator that is progressively downregulated in hippocampal neurons of AD patients and 5 × FAD mice, correlating with spatial memory deficits. Reducing SIK2 levels exacerbates cognitive impairment and amyloid-β (Aβ) plaque burden in mice, whereas restoring SIK2 levels mitigates these deficits, restores LTP amplitude, reverses synaptic ultrastructural pathology, and reduces Aβ deposition. Mechanistically, SIK2 enhances autophagic flux by phosphorylating GABARAPL2 at Ser72, a modification essential for autophagosome–lysosome fusion. Remarkably, hippocampal delivery of the phosphorylation-mimetic GABARAPL2-S72E mutant replicated the beneficial effects of SIK2, alleviating Aβ pathology and synaptic dysfunction in 5 × FAD mice. In contrast, the non-phosphorylatable S72A mutant failed to show any protective effects.

**Conclusions:**

These findings establish the SIK2–GABARAPL2 axis as a novel signaling cascade governing autophagic flux through lysosomal fusion competence. Dysfunction in this axis contributes to Aβ deposition in AD, offering new insights into the pathogenic mechanisms underlying autophagosome–lysosome fusion in AD and highlighting its potential as a therapeutic target.

**Graphical Abstract:**

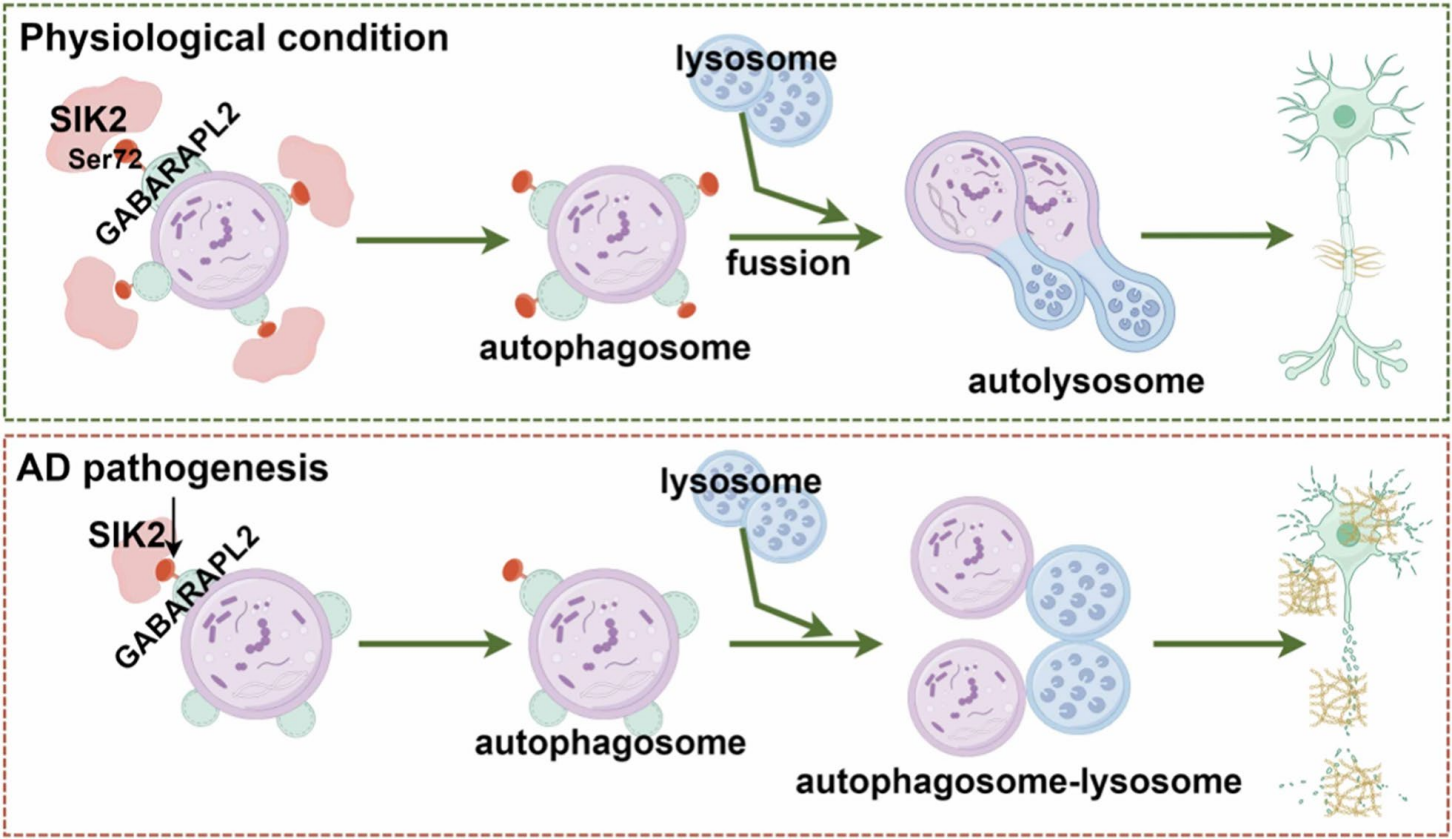

**Supplementary Information:**

The online version contains supplementary material available at 10.1186/s40035-025-00514-4.

## Background

Alzheimer’s disease (AD) is the most common neurodegenerative disorder, characterized by progressive cognitive decline, synaptic dysfunction, and neuropathological hallmarks such as amyloid-β (Aβ) plaques and neurofibrillary tangles composed of hyperphosphorylated tau protein [1–3]. Existing pathogenic models highlight the critical imbalance between Aβ generation and clearance, in which inadequate cerebral Aβ removal mechanisms serve as a key driver of disease progression [4–8]. This has driven research to prioritize therapeutic strategies to enhance Aβ clearance from the central nervous system in AD intervention studies [9–12].

The autophagy–lysosome pathway, which encompasses autophagy and subsequent lysosomal degradation, represents a vital quality control mechanism for eliminating misfolded proteins [13]. Impaired autophagosome–lysosome fusion leads to abnormal autophagic vacuole accumulation in neurons, while lysosomal acidification defects and reduced cathepsin activity impair Aβ degradation in AD [14–20]. Postmortem brain tissues from AD patients also show increased hippocampal levels of p62/SQSTM1—a marker of autophagic impairment—that correlate with disease severity [21, 22]. These observations indicate that restoring autophagic flux and lysosomal function is a promising therapeutic strategy for reducing Aβ pathology and preserving neuronal integrity in AD.

GABA type A receptor-associated protein-like 2 (GABARAPL2), a member of the ATG8 protein family, plays a critical role in autophagosome maturation and autophagosome–lysosome fusion—a key step in cargo degradation [23–26]. Post-translational modifications, particularly phosphorylation, dynamically regulate activities of ATG8 family proteins, thereby fine-tuning the autophagic flux [27–34]. For example, kinase-mediated phosphorylation of specific GABARAPL2 residues modulates its interaction with autophagy receptors and lysosomal membrane proteins, promoting autophagic vesicle docking and fusion [29, 32–34]. Additionally, age-related changes in kinase activity associated with the autophagy-lysosome pathway have been observed in neurodegenerative diseases [27–31]. However, the specific kinases involved in this regulatory process in neurons and their potential disruption in AD remain unclear.

Salt-inducible kinase 2 (SIK2), a key member of the AMP-activated protein kinase (AMPK) family, has emerged as a potential regulator of cellular metabolism and neuronal function. In peripheral tissues, SIK2 regulates autophagic flux by modulating TFEB (transcription factor EB) and mTOR pathways, thereby influencing lysosomal biogenesis—a mechanism critical for glucolipid metabolic reprogramming and cancer progression [35–37]. SIK2 enhances neuronal survival post-ischemia by activating the TORC1–CREB signaling axis, which boosts stress adaptation and mitochondrial autophagy [38, 39]. In depression models, inhibition of SIK2 (e.g., with ARN-3236) restores neurotrophic signaling and alleviates depressive behaviors through the hippocampal CRTC1–CREB–BDNF (brain-derived neurotrophic factor) cascade [40–44]. These findings identify SIK2 as a key regulator of autophagic flux and a potential therapeutic target for neuropsychiatric disorders. However, the mechanisms underlying this regulation and the role of SIK2 in AD-related pathologies remain unclear.

In this study, we sought to investigate whether SIK2-mediated phosphorylation contributes to autophagic dysfunction in AD. Using a combination of in vitro and in vivo models, this study aims to: (1) characterize SIK2 expression patterns during AD progression and their cognitive relevance; (2) identify specific ATG8 family members as potential substrates of SIK2-mediated phosphorylation; and (3) determine the functional consequences of this regulatory mechanism on autophagosome-lysosome fusion, Aβ clearance, and synaptic maintenance. Our findings provide new insights into the molecular mechanisms connecting kinase signaling with proteostatic failure in AD, offering potential avenues for therapeutic development.

## Materials and methods

### Animal models

5 × FAD transgenic mice (stock no. 034848-JAX, Bar Harbor, ME), which co-express human Amyloid precursor protein/Presenilin 1 (APP/PS1) with five familial AD mutations (Thy-1 promoter: *APP* K670N/M671L, I716V, V717I; *PS1* M146L/L286V), were maintained on a C57BL/6 background via crossing with WT mice (GemPharmatech, Nanjing, China). Genotypes were determined by PCR analysis of tail DNA as previously described [45]. Mice were group-housed (≤ 5 per cage) in Individual Ventilated Cages (IVC) systems (Tecniplast, Buguggiate, Italy) under controlled conditions (22 ± 1 °C, 12-h light/dark cycle) with ad libitum access to food and water. All animal protocols and procedures were approved by the Institutional Animal Care and Use Committee of Fujian Medical University (IACUC FJMU 2022–0859).

### Bioinformatics analysis

Human postmortem temporal cortex mRNA expression profiles were obtained from the Gene Expression Omnibus (GEO, https://www.ncbi.nlm.nih.gov/geo/; dataset GSE118553), comprising data from 167 AD patients (69 males, aged 66–105; 98 females, aged 61–98) and 100 non-dementia controls (55 males, aged 41–95; 45 females, aged 43–92). The dataset was generated using the Illumina HumanHT-12 V4.0 expression beadchip (GPL10558 platform).

### Stereotaxic virus injection

Six-month-old wild-type (WT) and 5 × FAD mice were anesthetized with 3% isoflurane and secured in a stereotaxic frame (RWD Instruments, Shenzhen, China). Bilateral hippocampal injections targeting CA1 (AP: − 1.9 mm, ML: ± 1.45 mm, DV: − 1.25 mm) and dentate gyrus (DV: − 1.85 mm) were performed using a NanoFil syringe (World Precision Instruments, Sarasota, FL). A total of 500 nL of virus was infused into each location over 10 min at ~ 50 nL/min. For SIK2 knock-down, the mice were stratified by sex into four cohorts: WT-control (scramble AAV, *n* = 13 males/12 males), WT-shSIK2 (*n* = 12 males/10 males), 5 × FAD-control (*n* = 10 males/9 females), and 5 × FAD-shSIK2 (*n* = 10 males/8 females). For SIK2 overexpression, the mice were stratified by sex into four cohorts: WT-control (scramble AAV, *n* = 11 males/10 females), WT-SIK2 (*n* = 8 males/14 females), 5 × FAD-control (*n* = 10 males/9 females), and 5 × FAD-SIK2 (*n* = 10 males/9 females). For overexpression of GABARAPL2 mutants, the mice were stratified by sex into five cohorts: WT-control (scramble AAV, *n* = 6 males/6 females), 5 × FAD-control (*n* = 5 males/5 females), 5 × FAD-GABARAPL2 (*n* = 6 males/4 females), 5 × FAD-GABARAPL2 (S72A) (*n* = 6 males/4 females), and 5 × FAD-GABARAPL2 (S72E) (*n* = 5 males/5 females). The following vectors were used: SIK2 knockdown virus (pAAV2/9-hSyn-EGFP-3FLAG-miR30shRNA(SIK2)-WPRE), control virus (pAAV2/9-hSyn-MCS-EGFP-3FLAG-miR30(shRNA)-WPRE), SIK2 overexpression virus (pAAV2/9-hSyn-EGFP-P2A-HA-SIK2-WPRE), control virus (pAAV2/9-hSyn-EGFP-P2A-WPRE), GABARAPL2 variant overexpression virus (pAAV2/9-hSyn-EGFP-P2A-HA-GABARAPL2-WPRE, pAAV2/9-hSyn-EGFP-P2A-HA-GABARAPL2-72E-WPRE, pAAV2/9-hSyn-EGFP-P2A-HA-GABARAPL2-72A-WPRE) and control virus (pAAV2/9-hSyn-EGFP-P2A-WPRE). All were designed, validated, and synthesized by OBiO Technology (Shanghai, China). Viral expression was validated by Western blot and qPCR prior to behavioral testing at 4 weeks post-injection. After injection, the cannula was left in place for 5 min to allow for complete spread of the virus, and then slowly withdrawn from the tissue. After 2 h of careful monitoring, the mice were returned to their cages. Then behavioural, biochemical and morphological analyses were performed.

### Electrophysiology

Mice were anesthetized with 3% isoflurane and decapitated. Brains were rapidly dissected in ice-cold, oxygenated (95% O₂/5% CO₂) artificial cerebrospinal fluid (containing in mmol/L: 124 NaCl, 3 KCl, 1.25 NaH₂PO₄, 26 NaHCO₃, 2 CaCl₂, 2 MgSO₄, 10 glucose). Coronal hippocampal slices (400 μm) were prepared using a Leica VT1200S vibratome, incubated at 32–34 °C for 30 min, and then equilibrated at room temperature for 1 h. Long-term potentiation (LTP) was assessed in the CA1 stratum radiatum using a Multiclamp 700B amplifier (Molecular Devices, San Jose, CA). Synaptic responses were evoked by stimulating Schaffer collateral axons. Baseline field excitatory postsynaptic potentials (fEPSPs) were recorded for 20 min at 30% maximal response intensity. LTP was induced by high-frequency stimulation (5 bursts at 5 Hz, repeated 5 times at 10 s intervals, 4 pulses at 100 Hz for each burst) and monitored for 60 min. Data were normalized to baseline and analyzed using pCLAMP 10.7 (fEPSP slope 10%–90% rise time). Slices with < 10% baseline drift were excluded.

### Transmission electron microscopy (TEM)

Tissues were fixed in 3% glutaraldehyde/1.5% paraformaldehyde in 0.1 mol/L PBS (pH 7.4) at 4 °C for 24–72 h, followed by post-fixation with 1% osmium tetroxide/1.5% potassium ferrocyanide for 1.5 h. After PBS rinsing, samples were en bloc stained with 2% uranyl acetate in 70% ethanol for 2 h, and then dehydrated via an ethanol-acetone gradient (50%, 70%, 90%, 100%). Tissues were embedded in epoxy resin 618 and polymerized at 60 °C for 48 h. Ultrathin sections (80 nm) were cut using a Leica UC7 ultramicrotome, double-stained with uranyl acetate (5 min) and lead citrate (5 min), and imaged under a FEI Tecnai G2 TEM (FEI, Hillsboro, OR) operated at 120 kV. Image acquisition was performed using a Gatan Orius SC1000 CCD camera.

### Morris water maze (MWM) test

Spatial learning and memory was assessed in a circular pool (120 cm diameter, 50 cm height) containing opaque water (22 ± 1 °C, 35-cm deep). A transparent escape platform (6-cm diameter) was placed 1.5 cm below the water surface in the target quadrant, with distinct geometric visual cues around the pool. Mice underwent four trials daily (60-s maximum per trial) for six consecutive days, starting from randomized entry points (N/E/S/W) using Vorhees-Williams pseudorandomization. Animals that failed to locate the platform within 60 s were guided to the platform and allowed 15 s for spatial orientation. Escape latency was averaged across daily trials. Probe testing was conducted 24 h after the training. The platform was removed, and swimming trajectories were recorded for 60 s using EthoVision XT 15 (Noldus, Wageningen, Netherlands), with analysis focusing on: target quadrant occupancy (%), platform-crossing frequency and the escape latency. Mice with impaired swimming ability (< 10 cm/s average speed) or thigmotaxis (> 80% periphery time) were excluded. Testing occurred under controlled illumination (50 lx) during the light phase.

### Quantitative polymerase chain reaction (qPCR)

Total RNA was extracted using the TRIzol Reagent (Invitrogen, Carlsbad, CA), and cDNA was synthesized using the One-Step Kit (Vazyme, R333-01, Nanjing, China). qPCR was performed on a Step One Plus Real-Time PCR System (Applied Biosystems, Foster City, CA) using Taq Pro Universal SYBR qPCR Master Mix (Vazyme, Q712-03) with the following primers: SIK2 forward: 5'-CTGCTGGCAACATGGTGTG-3', reverse: 5'-GGGAGAGTTGGTCCATCAAAAG-3'. Amplification efficiency (95%–105%) was validated via standard curves. Reactions were conducted in triplicate under the following conditions: 50 °C for 2 min, 95 °C for 10 min, and 40 cycles of 95 °C for 15 s and 60 °C for 1 min. Gene expression was quantified using the 2^−ΔΔCt^ method, with β-actin as a normalization control.

### Brain tissues/cell lysates and immunoblotting

Mice were deeply anesthetized and transcardially perfused with saline, after which hippocampal and cortical tissues were dissected. Tissues were homogenized in the RIPA buffer (Abcam, #ab156034, Cambridge, UK) containing protease inhibitors (Millipore, #539131, Billerica, MA) using a sonicator. Lysates were centrifuged at 12,000 × *g* for 25 min at 4 °C, and the resulting supernatant was collected for total protein analysis. Protein concentrations were determined using the BCA assay (Beyotime, P0009, Shanghai, China). Equal amounts of protein (30 μg) were separated by 10% SDS-PAGE and transferred to PVDF membranes. After blocking with 5% BSA for 1 h, membranes were incubated with primary antibodies (Table S1) overnight at 4 °C. HRP-conjugated secondary antibodies (1:5000) were applied for 1 h at room temperature (RT). Signals were detected using an Ultra High Sensitivity ECL Kit (MedChemExpress, HY-K1005, Monmouth Junction, NJ) and visualized using an imaging system. Target band intensities were quantified using ImageJ software 1.46 (Bethesda, MD).

### Co-immunoprecipitation (Co-IP) assay

Tissue and cell lysates were prepared in the RIPA buffer containing protease inhibitors. For SIK2–ATG interaction studies, 1000 μg of lysate was pre-cleared with Protein A/G agarose beads (Thermo Scientific, #88802, Wilmington, DE) for 1 h at 4 °C. The cleared supernatant was incubated overnight with anti-SIK2- or control IgG-conjugated beads. For flag-tagged protein analysis, lysates were directly incubated with anti-flag magnetic beads (Beyotime, #P2155). Beads were washed four times with ice-cold lysis buffer, and the bound proteins were eluted in 2 × SDS loading buffer at 100 °C for 10 min. The eluted proteins were separated by Western blot analysis using target-specific antibodies.

### Glutathione S-transferase (GST) pull-down assay

SIK2 protein was expressed in vitro using the eukaryote cell free system, TNT T7 Quick coupled transcription/translation (Promega, L1170, Madison, WI). For in vitro binding assay, the SIK2 protein was incubated with GST (Proteintech, Ag0040, Wuhan, China) or GST-GABARAPL2 (Proteintech, Ag1155) protein which was immobilized on glutathione-conjugated Sepharose beads. The beads were then washed three times and boiled in the SDS-PAGE loading buffer for 5 min, followed by immunoblot analysis. For competitive binding, SIK2 protein was incubated with GST-GABARAPL2 and increasing concentrations of LC3-interacting region (LIR) 326–333 (FAAIYFLL) peptide (Sangon Biotech, P37903, Shanghai, China) and subjected to the GST pull-down assay.

### Tissue and cellular immunofluorescence (IF) assays

Mice were transcardially perfused with 4% paraformaldehyde (PFA) in PBS (pH 7.4). Brains were post-fixed in 4% PFA at 4 °C for 18 h, cryoprotected in 30% sucrose, and embedded in the OCT compound. Coronal sections (40 μm) were cut using a cryostat (Leica, CM1950, Nussloch, Germany) and stored in antifreeze solution. For cellular samples, N2a and N2a-APP cells were transfected with an mRFP-GFP-LC3B-expressing plasmid (Beyotime, D2816). Twenty-four hours post-transfection, cells cultured on coverslips were gently rinsed with PBS and fixed for 20 min at room temperature in 4% PFA in 0.01 mol/L PBS (7:3, *v*/*v*). Tissue sections and cells were incubated with glycine for 20 min, then blocked with 5% donkey serum and 0.3% Triton X-100 in TBS for 1 h at RT. Primary antibodies (Table S1) diluted in blocking buffer were applied overnight at 4 °C. After washes, Alexa Fluor-conjugated secondary antibodies and DAPI (CST, #4083, Danvers, MA) were applied for 10 min at RT. Sections were mounted with ProLong Gold Antifade Mountant (Invitrogen, P36934, Carlsbad, CA) and imaged using a Zeiss LSM 780 confocal microscope. Quantitative analysis of fluorescence intensity and colocalization was performed using the FIJI software (ImageJ 1.46, NIH, Bethesda, MD) with threshold-based masking.

### RNAscope combined with immunofluorescence

RNAscope was performed according to the manufacturer’s instructions (Advanced Cell Diagnostics, Hayward, CA) using a proprietary probe targeting SIK2 (ACD, 526421, RNAscope Probe—Mm-*SIK2*). Briefly, 20-μm-thick sections were prepared and subjected to the following sequential treatments: RNAscope® Hydrogen Peroxide for 10 min at room temperature to fully expose RNA targets, followed by protease digestion at 40 °C for 30 min. After each pretreatment step, sections were rinsed with water. Hybridization was carried out with the SIK2-specific probe at 40 °C for 2 h without a coverslip. After hybridization, sections underwent serial signal amplification steps (Amp 1–6) with wash buffer applied after each amplification reagent. Subsequent immunofluorescence staining was performed following standard protocols to enable multiplex target visualization.

### Enzyme-linked immunosorbent assay (ELISA) for Aβ_1–42_

Hippocampal tissues were homogenized in the RIPA buffer (25 mmol/L Tris–HCl, pH 7.4; 150 mmol/L NaCl; 1% NP-40) containing protease inhibitors using a sonicator (100 W, 1 s on/2 s off, 20 cycles). Lysates were centrifuged at 16,000 × *g* for 5 min at 4 °C to collect TBS-soluble Aβ_1–42_. Pellets were further extracted with 5 mol/L guanidine-HCl (4 h shaking at RT) to recover insoluble Aβ_1–42_. The soluble and insoluble fractions were diluted 1:10 in standard diluent buffer (Invitrogen, #KHB3441) to neutralize guanidine. Aβ_1–42_ levels were quantified using a human-specific ELISA kit (Invitrogen, #KHB3441) following the manufacturer’s protocol. Total protein concentration was determined using the BCA assay for data normalization.

### RNA-sequencing (RNA-seq) analysis

Total RNA from the mouse dorsal hippocampus was processed into strand-specific cDNA libraries after ribosomal RNA depletion. Paired-end sequencing (150 bp) was performed by Genedenovo Biotechnology (Guangzhou, China). High-quality reads were aligned to the GRCm36/mm10 genome using HISAT2, and gene expression was quantified as FPKM (fragments per kilobase of transcript per million fragments mapped) using the RSEM 1.2.19 package. Differentially expressed genes (DEGs) were identified using the DESeq2 software (fold change ≥ 1.2, false discovery rate [FDR] < 0.05). Functional enrichment analysis of DEGs was performed based on the Kyoto encyclopedia of genes and genomes (KEGG) pathway analysis using the clusterProfiler software (FDR < 0.05).

### Post-translational modification proteomics

Tissues were ground in liquid nitrogen in a denaturing lysis buffer (containing 8 mol/L urea, 1% protease inhibitors, 3 μmol/L trichostatin A, and 50 mmol/L nicotinamide) to suppress deacetylase activity, followed by sonication and centrifugation (12,000 × *g*, 10 min). The protein supernatant was quantified using the BCA assay, precipitated with 20% trichloroacetic acid, and subjected to tryptic digestion (enzyme-to-protein ratio, 1:50) after reduction and alkylation. Phosphopeptides were enriched using IMAC (immobilized metal-ion affinity chromatography) microspheres. The enriched peptides were separated on an Easy-nLC 1000 UHPLC system (Bruker Daltonics, Billerica, MA) with a 25-cm column and a 2%–90% acetonitrile gradient, then analyzed on a timsTOF Pro mass spectrometer (Bruker) in the dia-PASEF mode (100–1700 m/z, 22 PASEF scans, MS/MS mode). The data were processed using Spectronaut v18 against the UniProt/SwissProt human database (concatenated with a decoy reverse database) with fixed carbamidomethylation (Cys) and variable phosphorylation (Ser/Thr/Tyr) and acetylation (N-terminus/Lys) modifications. FDR < 1% was maintained at the protein, peptide, and Peptide-Spectrum Match (PSM) levels. Functional annotations (Gene Ontology, KEGG pathways, PFAMdomains) and enrichment analyses (Fisher’s exact test, *P* < 0.05) were performed. Protein–protein interaction networks were constructed using STRING (v11.5) (confidence score > 0.7). Sequencing and preliminary data processing were performed by Jingjie PTM BioLab (Hangzhou, China).

### Golgi-Cox staining and dendritic spine analysis

Mouse brain tissues were rinsed with PBS and immersed in pre-mixed Golgi-Cox solutions (Solution 1 & 2, Golgi Staining Kit, Hitobiotec, HTKNS1125, Kingsport, TN) for 15 days at RT in the dark, followed by incubation in Solution 3 at 4 °C for 4 days. Tissues were frozen in isopentane, embedded, and sectioned coronally (100 μm) using a cryostat (Leica CM1950). Sections were mounted on gelatin-coated slides, air-dried at RT for 72 h, and sequentially processed as follows: (1) ammonia developer (Solution 4/5/water mixture) for 10 min; (2) two distilled water rinses for 4 min each; (3) ethanol dehydration (50%, 75%, 95%, 100%) with four changes, 5 min each; and (4) xylene clearing three times for 5 min each. Slides were coverslipped with the resinous mounting medium. Dendritic morphology was imaged under brightfield illumination (T-PMT) using a Zeiss LSM 780 microscope with 10 × and 63 × oil immersion lenses. Dendritic complexity was quantified by Sholl analysis as previously described [30], and spine density was analyzed using the FIJI software.

### Statistical analysis

All data are presented as the mean ± SEM (standard error of the mean) from at least three independent experiments, each with three technical replicates. Statistical analyses were performed using GraphPad Prism 9.0. Data distribution was assessed using the Shapiro–Wilk normality test, and most data were normally distributed. Statistical differences were determined using unpaired two-tailed *t*-tests or one-way, two-way, or three-way analysis of variance (ANOVA) followed by Tukey’s post-hoc multiple comparisons test. *p*-value < 0.05 was considered statistically significant.

## Results

### Age-dependent downregulation of SIK2 in AD models and patients

To investigate the potential role of autophagic dysfunction in AD pathogenesis, we assessed autophagic activity in WT and 5 × FAD transgenic mice. Quantitative PCR showed no significant changes in the mRNA expression of autophagosome marker LC3B in 5 × FAD mice versus WT controls, while the mRNA expression of the cargo receptor p62 was significantly upregulated in the hippocampus (Fig. S1a). Western blot analysis confirmed concurrent increases in LC3B and p62 protein levels in the hippocampus of 5 × FAD mice (Fig. S1b, c), mirroring prior observations of autophagic vacuole accumulation in AD models [46, 47]. These data indicate defective autophagic flux and impaired autophagosome–lysosome fusion in AD.

The autophagosome–lysosome fusion is orchestrated by two functionally distinct modules: (i) the ATG8 conjugation system that primes autophagosomal membranes for docking, and (ii) the trafficking apparatus comprising RAB GTPases (e.g., RAB7A and RAB33B), SNAREs (e.g., VAMP8, STX17, SNAP29, and YKT6), and PLEKHM1 tethering protein [48–51]. We therefore dissected which module underpins the fusion block in AD. Further analysis identified elevated *Vamp8* mRNA in 5 × FAD mice (Fig. S1d); however, bioinformatic analysis of a human postmortem temporal cortex transcriptomic dataset (GSE118553, *n* = 267) revealed no significant alteration in the expression of autophagosome-trafficking genes (*VAMP8*, *STX17*, *SNAP29*, *YKT6*, *RAB7A*, *RAB33B* and *PLEKHM1*) between AD patients (*n* = 167) and non-demented controls (*n* = 100) (Fig. S1e). Collectively, these findings argue against a primary defect in vesicular trafficking and instead implicate dysregulation of the ATG8 conjugation system as the central mechanism underlying autophagosome–lysosome fusion failure in AD.

**Fig. 1:**
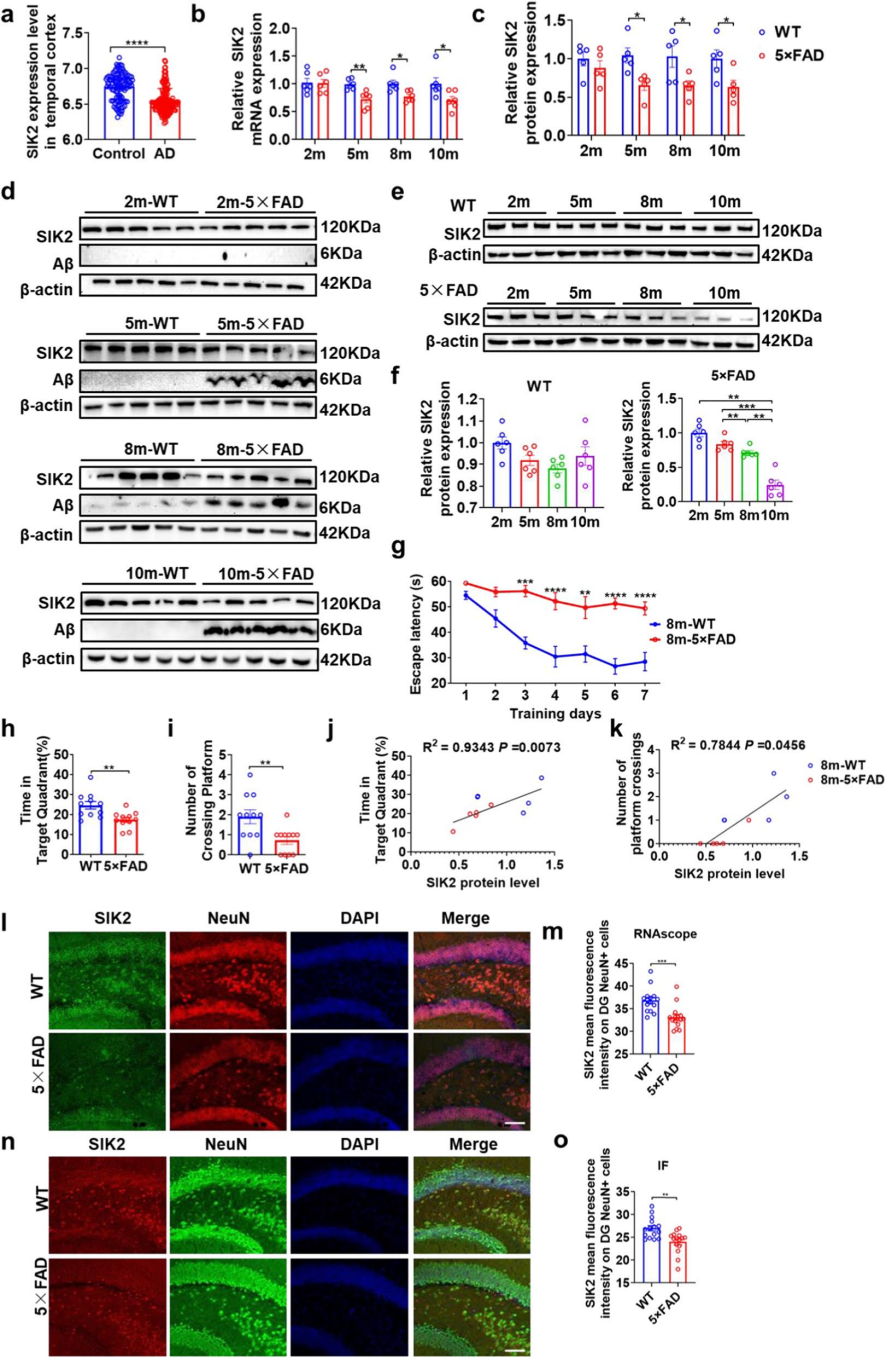
Age-dependent downregulation of SIK2 was observed in the middle-aged AD transgenic mouse models and AD patients. **a** Bioinformatics analysis revealed reduced SIK2 expression in the temporal cortex of AD patients compared to controls. **b** *Sik2* mRNA levels in the hippocampus of 2-, 5-, 8-, and 10-month-old WT and 5 × FAD mice (*n* = 6/group), assessed by qPCR. **c** Quantification of SIK2 protein levels in the hippocampus of 2-, 5-, 8-, and 10-month-old WT and 5 × FAD mice (*n* = 5/group), assessed by western blot. **d** Representative immunoblots of SIK2 in the dorsal hippocampus of 2-, 5-, 8-, and 10-month-old WT and 5 × FAD mice. **e, f** Representative immunoblots and quantification of SIK2 in the hippocampus of 2-, 5-, 8-, and 10-month-old WT and 5 × FAD mice (*n* = 3/group). **g, h** MWM performance of WT and 5 × FAD mice. Escape latency during training trials (1–7 days) (**g**). Percentage of time spent in the target quadrant during the probe trial (day 8) (**h**). **i** Number of platform crossings during the probe trial (day 8). **j, k** Linear regression analysis was performed to assess the relationship between hippocampal SIK2 protein levels and cognitive performance (percentage of time in the target quadrant (**j**) and number of platform crossings (**k**)) in WT (*n* = 5) and 5 × FAD mice (*n* = 5). The coefficient of determination (*R*^2^) and the *P* value from the F-test of the overall fit are shown on the graphs. The solid line represents the line of best fit. **l** SIK2-specific RNA probes combined with NeuN immunofluorescence in the dentate gyrus (DG) of WT and 5 × FAD mice. Scale bar, 50 μm. **m** Quantification of SIK2 intensity in DG NeuN⁺ cells (*n* = 3/group). **n** Double-label immunofluorescence showing SIK2 (red) and NeuN (green) colocalization in the DG region of WT and 5 × FAD mice. Scale bar, 50 μm. **o** Quantification of SIK2 intensity in DG NeuN⁺ cells (*n* = 3/group). Data are expressed as mean ± SEM. Statistical significance was calculated by unpaired Mann–Whitney test (**i**), unpaired two-tailed *t*-test (**a, h, m, o**), linear regression analysis (**j, k**), one-way ANOVA (**b, c, f**), and two-way ANOVA (**g**) followed by the Tukey’s post-hoc test. *P < 0.05, **P < 0.01, ***P < 0.001, ****P < 0.0001

Proteomic analyses indicate that post-translational modifications, particularly kinase-mediated phosphorylation within the ATG8 conjugation system, govern the autophagosome–lysosome fusion [30, 52]. We screened a panel of fusion-associated kinases (including STK3, STK4, NEK9, ULK1-3, TBK1, PIK3C3/R4, and SIK2) and found that *Sik2* mRNA was selectively and significantly reduced in the hippocampus of 5 × FAD mice (Fig. 1b), while other kinases remained unchanged (Fig. S1f). Bioinformatics analysis of human AD cohorts (GSE118553) confirmed the downregulation of *SIK2* and *ULK1* genes, along with elevated *STK3* mRNA expression (Fig. 1a, Fig. S1g). Given the consistent downregulation of *SIK2* in both murine and human AD models, we next interrogated its functional contribution to the autophagy–lysosomal machinery in AD.

We quantified SIK2 levels in the hippocampus of 5 × FAD mice at various ages. SIK2 mRNA and protein levels showed no significant changes at 2 months, but decreased markedly at 5, 8, and 10 months in 5 × FAD mice (Fig. 1b–d). Comparison between ages revealed that the SIK2 protein levels in 5 × FAD mice declined significantly by 8 months of age and further worsened by 10 months, while the WT mice showed no significant changes across ages (Fig. 1e, f). MWM at 8 months showed that the 5 × FAD mice exhibited longer escape latencies during training trials (days 3–7) and in the probe trial (day 8) compared to the age-matched WT mice (Fig. 1g). Additionally, the 5 × FAD mice spent less time in the target quadrant (Fig. 1h) and fewer crossings of the platform location (Fig. 1i). These results indicate significant spatial learning and memory deficits in 8-month-old 5 × FAD mice. Notably, SIK2 protein levels correlated positively with the percentage of time spent in the target quadrant (*R*^2^ = 0.9343, *P* = 0.0073) (Fig. 1j) and the number of platform crossings (*R*^2^ = 0.7844, *P* = 0.0456) (Fig. 1k).

We next examined the distribution of SIK2 gene expression among cell types. Immunofluorescence and western blotting indicated that SIK2 was ubiquitously expressed but significantly enriched in neurons (Fig. S2a–d). To assess neuron-specific SIK2 expression in 5 × FAD mice, we quantitatively analyzed hippocampal subregions in 8-month-old mice using *Sik2*-specific RNA probes combined with NeuN immunofluorescence. In the dentate gyrus (DG), 5 × FAD mice showed a significant reduction in *Sik2* staining intensity within NeuN⁺ neurons compared to control mice (Fig. 1l, m). This attenuation extended to CA1 and CA3 regions (Fig. S2e, f). IF staining with anti-SIK2 and anti-NeuN antibodies confirmed reduced SIK2 fluorescence intensity within NeuN⁺ neurons in the DG (Fig. 1n, o), CA1 and CA3 regions of 5 × FAD mice (Fig. S2g, h), demonstrating a broad hippocampal SIK2 deficit. These results suggest that the spatial cognitive decline in the 5 × FAD mice is directly related to age-associated decrease of SIK2 expression in the hippocampus.

### SIK2 alleviates the cognitive impairment and enhances synaptic plasticity in middle-aged 5 × FAD mice

To evaluate the role of SIK2 in the hippocampus-associated spatial learning and memory, we downregulated SIK2 in the dorsal hippocampus of 6-month-old WT and age/sex-matched 5 × FAD mice using an adeno-associated shSIK2 virus (Fig. S3a). The knockdown efficiency was confirmed by Western blotting and qPCR (Fig. S3b–d). MWM revealed that the 5 × FAD-control mice exhibited significantly longer escape latencies on training days 5–7 than WT-control mice. Notably, the escape latencies of the 5 × FAD-control mice were significantly shorter than that of the 5 × FAD-shSIK2 mice on day 7 (Fig. S3e). In the probe test, both 5 × FAD-control and 5 × FAD-shSIK2 groups showed fewer platform crossings than the WT-control and WT-shSIK2 groups. *Sik2* knockdown further reduced platform crossings compared to respective control groups (Fig. S3f). Additionally, the 5 × FAD-control and 5 × FAD-shSIK2 mice spent less time in the target quadrant than WT-control and WT-shSIK2 mice. The 5 × FAD-shSIK2 mice spent less time in the target quadrant than 5 × FAD-control mice (Fig. S3g). Consistently, the 5 × FAD-control mice had longer escape latencies than the WT-control mice, and *Sik2* knockdown further increased the escape latencies in 5 × FAD mice (Fig. S3h). No significant difference in the swimming speed was observed among the groups (Fig. S3i). These results demonstrate that *Sik2* knockdown in the dorsal hippocampus further impairs learning and memory in middle-aged 5 × FAD mice.

**Fig. 2:**
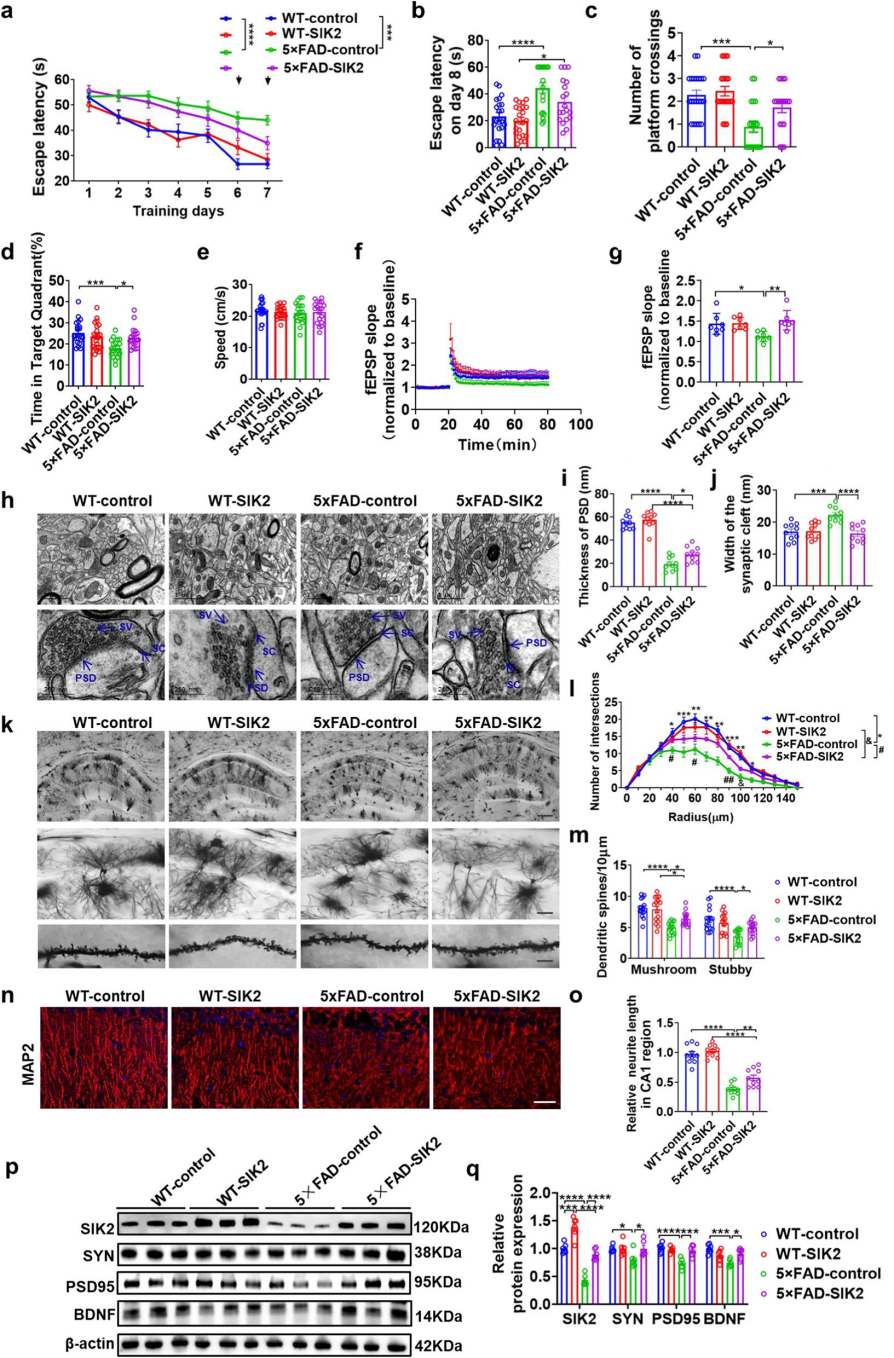
SIK2 alleviates the cognitive impairment and enhances the synaptic plasticity in middle-aged 5 × FAD mice. **a–e** MWM performance of WT-control, WT-SIK2, 5 × FAD-control, and 5 × FAD-SIK2 mice. Escape latency during training trials (1–7 days) (**a**) and probe trial (day 8) (**b**). Platform crossings (**c**), percentage of time in the target quadrant (**d**), and swimming speed (**e**) during the probe trial (day 8). Sample sizes: *n* = 21 (WT-control), *n* = 22 (WT-SIK2), *n* = 19 (5 × FAD-control), *n* = 19 (5 × FAD-SIK2). **f, g** Long-term potentiation (LTP) recordings in hippocampal CA1 regions. High-frequency stimulation (HFS) was applied, and fEPSP amplitudes were quantified during the last 10 min (**g**). Sample sizes: *n* = 3 mice, 7 slices per group. **h** Ultrastructural analysis of synapses in hippocampal CA1 region via TEM. sv, synaptic vesicle; sc, synaptic cleft; PSD, postsynaptic density. Scale bars: 1 µm (top images), 250 nm (bottom images). **i, j** Quantitative analysis of PSD thickness and SC width (*n* = 2 mice, 5 images per mouse). **k-m** Dendritic morphology of CA1 pyramidal neurons. Representative images (**k**) and Sholl analysis of branch intersections (**l-m**). Scale bars, 500 µm (upper), 50 µm (middle), 10 µm (lower). Sample size: *n* = 7 dendrites from 3 mice per group. **n, o** MAP2 immunofluorescence staining in the CA1 region. Representative images (**n**) and quantitative analysis (**o**). Scale bar, 50 µm. **p, q** Western blot analysis of SIK2, SYN, PSD95, and BDNF in dorsal hippocampal lysates. Representative immunoblots (**p**) and quantitative analyses (**q**) (*n* = 6 per group). Data are expressed as mean ± SEM. Statistical significance was calculated by two-way ANOVA (**b, c-g**, **i, j, l, m, o, q**), and three-way ANOVA (**a**) followed by the Tukey’s post-hoc test, and Scheirer-Ray-Hare test followed by the Dunn’s post-hot test (**c**). *P < 0.05, **P < 0.01, ***P < 0.001, ****P < 0.0001, ^#^*P* < 0.05, ^##^*P* < 0.01, ^&^*P* < 0.05

We also injected adeno-associated virus expressing SIK2 in the dorsal hippocampus of 6-month-old 5 × FAD mice (Fig. S4a). Western blot, qPCR and immunofuorescence analyses showed that SIK2 expression in the hippocampal neurons of both 5 × FAD and WT mice was significantly upregulated (Fig. S4b–f). Next, we examined whether SIK2 upregulation can rescue the impaired spatial learning and memory in the MWM test. Similarly, on days 6–7 of training, the 5 × FAD-control mice showed significantly prolonged escape latency compared to the WT-control on days 6–7 of training and on day 8 in the probe test (Fig. 2a, b). SIK2 overexpression rescued this deficit in 5 × FAD mice (Fig. 2b). Both the number of platform crossings and the target quadrant occupancy in the 5 × FAD-control group were lower than those of the WT-control group. SIK2 overexpression significantly ameliorated these deficits in 5 × FAD mice, restoring both parameters to levels comparable to the WT groups, with no noticeable difference between WT-control and WT-SIK2 groups (Fig. 2c, d). There was no significant difference in the swimming speed between these groups (Fig. 2e). These results indicate that SIK2 upregulation alleviates the cognitive impairment in the middle-aged 5 × FAD mice.

Electrophysiological recordings demonstrated impaired LTP maintenance at the CA3-CA1 synapses in 5 × FAD-control mice, with a significant reduction in the LTP amplitude at 60 min post-tetanic stimulation compared to the WT-controls. This reduction was rescued by SIK2 upregulation (Fig. 2f, g). Ultrastructural analysis by TEM revealed pathological synaptic alterations in 5 × FAD-controls, as evidenced by diminished postsynaptic density (PSD) thickness and broadened synaptic cleft (SC). However, SIK2 overexpression significantly ameliorated these synaptic ultrastructure impairments compared to the 5 × FAD-control group, resulting in thicker PSD and narrower SC (Fig. 2h–j). Sholl analysis of Golgi-stained hippocampal neurons demonstrated decreased dendritic arborization in 5 × FAD-controls, with reduced intersections at 40–100 μm radii and decreased mature dendritic spine density. SIK2 overexpression reversed these deficits, increasing dendritic complexity and spine density to be comparable to WT controls (Fig. 2k–m). Quantification of MAP2 (microtubule-associated protein 2)-positive dendrites demonstrated significant shortening of dendritic length of CA1 neurons in 5 × FAD-controls versus WT-controls, which was rescued by SIK2 overexpression (Fig. 2n, o). Western blot analysis showed significant downregulation of synaptophysin (SYN), postsynaptic density protein 95 (PSD95), and BDNF in 5 × FAD-controls versus WT-controls, which was reversed by SIK2 upregulation and further reduced by SIK2 knockdown (Fig. 2p, q; Fig. S5a, b). Together, these results show that SIK2 promotes synaptic plasticity in the middle-aged 5 × FAD mice.

### SIK2 reduces Aβ deposition in 5 × FAD mice

**Fig. 3:**
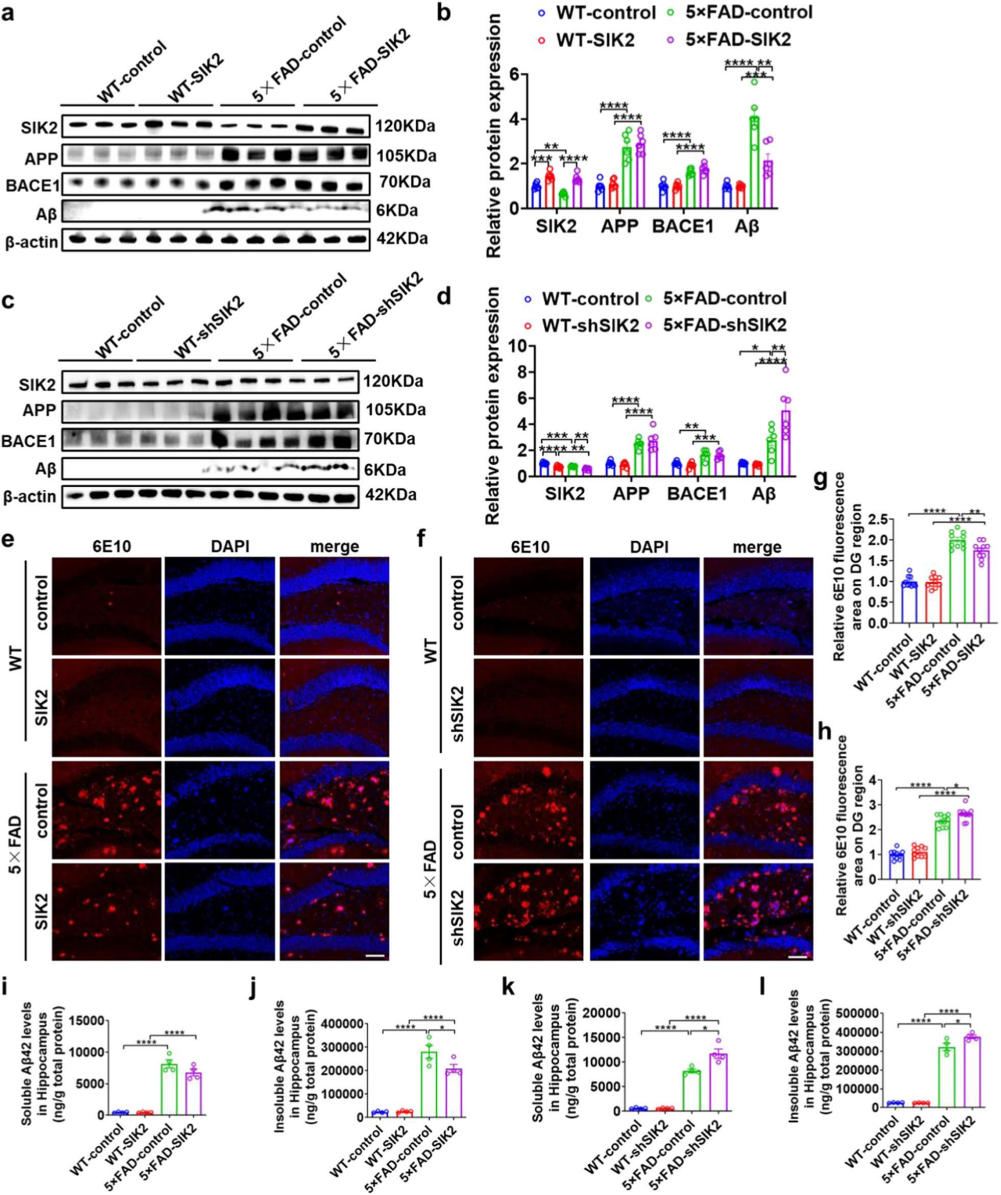
SIK2 reduces Aβ deposition in 5 × FAD mice. **a, b** Representative immunoblots and quantitative analyses of SIK2, Aβ (6E10), APP, and BACE1 in the dorsal hippocampus of WT-control, WT-SIK2, 5 × FAD-control, and 5 × FAD-SIK2 mice (*n* = 6/group). **c, d** Representative immunoblots and quantitative analyses of SIK2, Aβ (6E10), APP, and BACE1 in the dorsal hippocampus of WT-control, WT-shSIK2, 5 × FAD-control, and 5 × FAD-shSIK2 mice (*n* = 6/group). **e, g** Immunofluorescence staining for Aβ (6E10) in the dentate gyrus (DG) of WT-control, WT-SIK2, 5 × FAD-control, and 5 × FAD-SIK2 mice. Representative images (**e**) and quantification (**g**) (*n* = 3/group). Scale bar, 50 µm. **f, h** Immunofluorescence staining for Aβ (6E10) in the DG of WT-control, WT-shSIK2, 5 × FAD-control, and 5 × FAD-shSIK2 mice. Representative images (**f**) and quantification (**h**) (*n* = 3/group). Scale bar, 50 µm. **i, j** ELISA quantification of soluble and insoluble Aβ_1–42_ levels in hippocampal homogenates of WT-control, WT-SIK2, 5 × FAD-control, and 5 × FAD-SIK2 mice (*n* = 4/group). **k, l** ELISA quantification of soluble and insoluble Aβ_1–42_ levels in hippocampal homogenates of WT-control, WT-shSIK2, 5 × FAD-control, and 5 × FAD-shSIK2 mice (*n* = 4/group). Data are expressed as mean ± SEM. Statistical significance was calculated by two-way ANOVA (**b-d**, **g-l**) followed by the Tukey’s post-hoc test. *P < 0.05, **P<0.01, ***P < 0.001, ****P < 0.0001

We investigated the effect of SIK2 on AD-related Aβ pathology. Western blot analysis revealed elevated total Aβ protein levels in 5 × FAD-control mice. This elevation was reduced by SIK2 overexpression (Fig. 3a, b) while increased by SIK2 knockdown (Fig. 3c, d). Aβ peptide production is initiated by BACE1-mediated cleavage of amyloid precursor protein (APP). However, SIK2 overexpression or knockdown did not affect APP or BACE1 levels in either WT or 5 × FAD mice (Fig. 3a–d). Immunofluorescence analysis showed that the 5 × FAD-control mice had significantly higher Aβ plaque burdens than WT-controls (Fig. 3e–h). Notably, SIK2 overexpression in 5 × FAD reduced Aβ plaque deposition (Fig. 3e, g), while SIK2 knockdown in 5 × FAD mice increased plaque accumulation (Fig. 3f, h). Consistently, ELISA results showed that both soluble and insoluble Aβ_42_ levels were higher in 5 × FAD-control mice than in WT-controls (Fig. 3i-l). SIK2 overexpression significantly reduced the soluble and insoluble Aβ_42_ levels (Fig. 3i, j), whereas SIK2 knockdown increased them in 5 × FAD mice (Fig. 3k, l). Overall, these results indicate that SIK2 reduces Aβ deposition primarily by enhancing intracellular Aβ clearance rather than by altering Aβ production via APP processing.

### SIK2 enhances the autophagic flux in AD models

**Fig. 4:**
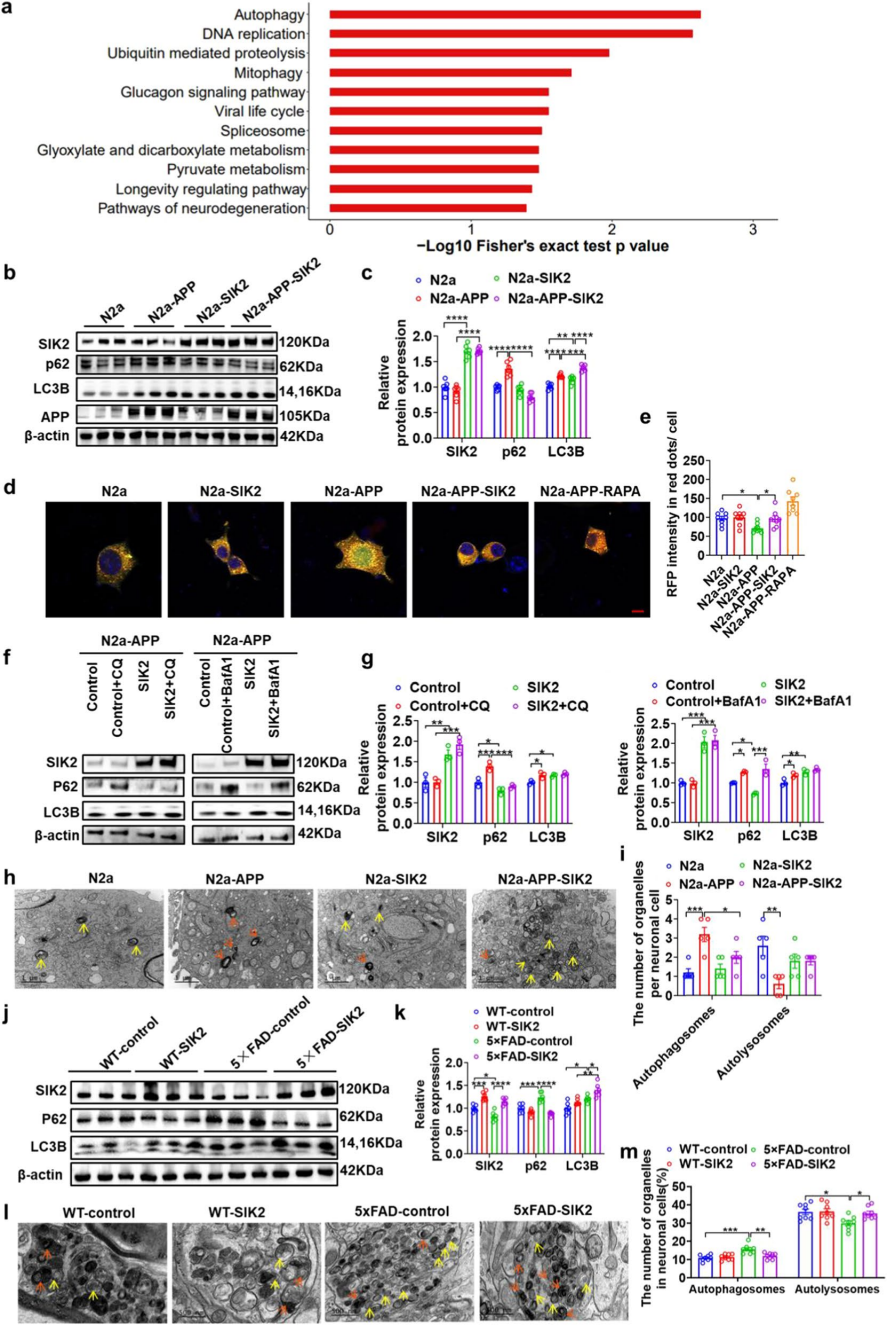
SIK2 enhances autophagic flux in AD models. **a** KEGG pathway enrichment analysis showing significant activation of autophagy-related pathways in N2a-APP-SIK2 versus N2a-APP cells (*P* = 0.0024, FDR < 0.05). **b, c** Representative immunoblots and quantitative analyses of SIK2, p62, LC3B, and APP in N2a, N2a-APP, N2a-SIK2, and N2a-APP-SIK2 cells (*n* = 6). **d, e** Detection of lysosomal acidification in N2a, N2a-SIK2, N2a-APP, N2a-APP-SIK2, and N2a-APP-RAPA cells by mRFP-GFP-LC3 tandem fluorescence. Autophagosomes show double mRFP ^+^GFP ^+^  signals (yellow spots), and functional autophagosomes showed mRFP ^+^ GFP^-^ signals (red spots). Representative images (**d**) and quantization (**e**) (*n* = 8–9/group). Scale bar, 10 μm. **f, g** Representative immunoblots and quantitative analyses of SIK2, p62, and LC3B in N2a-APP cells treated with chloroquine (CQ, 50 μmol/L, 12 h) or bafilomycin A1 (BafA1, 100 nmol/L, 12 h) (*n* = 3 per group). **h, i** TEM images of autophagosomes (orange arrows) and autolysosomes (yellow arrows) in N2a, N2a-APP, N2a-SIK2, and N2a-APP-SIK2 cells. Scale bars, 1 µm. Quantitative analysis of autophagic vacuoles (**i**). **j, k** Representative immunoblots and quantitative analyses of SIK2, p62, and LC3B in WT-control, WT-SIK2, 5 × FAD-control, and 5 × FAD-SIK2 mice (*n* = 6/group). **l, m** TEM images of autophagosomes (orange arrows) and autolysosomes (yellow arrows) in WT-control, WT-SIK2, 5 × FAD-control, and 5 × FAD-SIK2 mice. Scale bars, 500 nm. Quantitative analysis of autophagic vacuoles (**m**). Data are expressed as mean ± SEM. Statistical significance was calculated by unpaired two-way ANOVA (**c, e, g, i, k, m**) followed by the Tukey’s post-hoc test. *P < 0.05, **P < 0.01, ***P < 0.001, ****P < 0.0001

To elucidate how SIK2 regulates Aβ deposition, we performed phosphoproteomic profiling in the N2a-APP and N2a-APP-SIK2 cells. Principal component analysis (PCA) demonstrated a clear separation between the two groups, indicating that SIK2 overexpression induces distinct phosphorylation patterns (Fig. S6a). Consistently, heatmap analysis revealed distinct protein expression patterns between the two groups (Fig. S6b), with 621 differentially expressed proteins identified (fold change > 1.5, *P* < 0.05), including 253 upregulated and 368 downregulated targets in the SIK2-overexpressing cells (Fig. S6c). KEGG pathway enrichment analysis highlighted significant activation of the autophagy pathway (*P* = 0.0024, FDR < 0.05) (Fig. 4a).

Further mechanistic insights were obtained through biomarker analysis. The LC3B protein level was elevated in N2a-APP cells compared to N2a cells and increased further in N2a-APP-SIK2 cells. Conversely, p62 accumulated in the N2a-APP cells but was reduced by SIK2 overexpression, indicating enhanced autophagic initiation and restored flux (Fig. 4b, c). To directly monitor the autophagic flux dynamics, we performed the mRFP-GFP-LC3 tandem fluorescence assay. This assay leverages lysosomal acidification-induced GFP quenching: autophagosomes display dual mRFP^+^GFP^+^ signals (yellow puncta), whereas functional autolysosomes exhibit mRFP^+^GFP^−^ signals (red puncta) post-fusion. Quantitative analysis demonstrated significantly reduced red puncta in N2a-APP cells versus controls, confirming impaired fusion. Importantly, SIK2 overexpression in N2a-APP cells robustly increased autolysosome formation, with rapamycin treatment serving as a positive control (Fig. 4d, e). Co-IP assays demonstrated that SIK2 restored the LC3B–LAMP1 interaction in N2a-APP cells, a critical step for autophagosome–lysosome fusion (Fig. S7a, b). Pharmacological inhibition clarified that SIK2 overexpression suppressed the p62 accumulation induced by the autophagosome–lysosome fusion blocker CQ, while having no effect on the accumulation induced by bafilomycin A1, a dual blocker for autophagosome–lysosome fusion and lysosomal acidification (Fig. 4f, g). This demonstrated that SIK2 enhanced the autophagic flux specifically at the autophagosome–lysosome fusion step, independent of lysosomal acidification. Ultrastructural analysis by TEM revealed excessive accumulation of double-membrane autophagosomes in N2a-APP cells, indicative of impaired lysosomal fusion. Notably, SIK2 overexpression reduced the autophagosome density and increased mature autolysosomes with single-limiting membranes and electron-dense contents (Fig. 4h, i), identifying autophagosome–lysosome fusion as a key regulatory point.

Consistent with the cellular findings, 5 × FAD-control mice exhibited autophagic impairment, evidenced by elevated hippocampal LC3B and p62 levels. SIK2 overexpression in 5 × FAD mice normalized p62 levels and sustained LC3B elevation. In contrast, SIK2 knockdown in 5 × FAD mice further increased p62 while maintaining the elevated LC3B (Fig. S5a, b). TEM analysis showed increased double-membrane autophagosomes and decreased single-membrane autolysosomes in 5 × FAD-control mice compared to WT-controls. SIK2 overexpression rescued this defect, reducing the number of autophagosomes and increasing the number of autolysosomes in 5 × FAD-SIK2 mice (Fig. 4l, m). Collectively, these results demonstrate that SIK2 upregulates the autophagic flux by promoting the autophagosome–lysosome fusion.

### SIK2 phosphorylates GABARAPL2 at Serine 72 to enhance autophagic flux

**Fig. 5:**
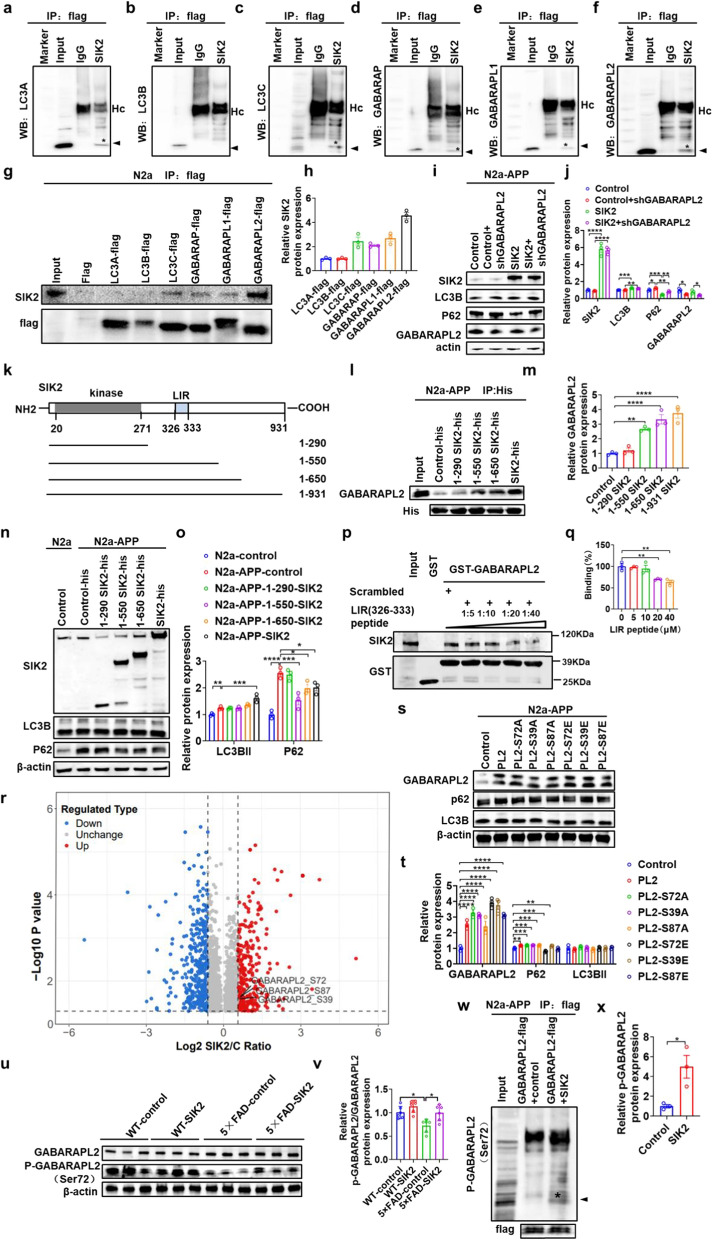
SIK2 phosphorylates GABARAPL2 at Serine 72 to enhance autophagic flux. **a-f** Co-IP analysis of interactions between SIK2 and LC3A (**a**), LC3B (**b**), LC3C (**c**), GABARAP (**d**), GABARAPL1 (**e**), or GABARAPL2 (**f**) in N2a cells overexpressing SIK2-flag, using anti-flag antibodies. Asterisks indicate co-precipitated ATG8 bands. Hc: IgG heavy chain. **g, h** Co-IP analysis of interactions between LC3A, LC3B, LC3C, GABARAP, GABARAPL1, or GABARAPL2 and SIK2 in N2a cells overexpressing respective flag-tagged ATG8 family members, using anti-flag antibodies. **i, j** Representative immunoblots and quantification of SIK2, p62, and LC3B in N2a-APP cells with GABARAPL2 knockdown and SIK2 overexpression (*n* = 3/group). **k** Schematic of SIK2 domain organization highlighting the kinase domain and LIR motif. Deletion mutants are illustrated below. **l, m** Co-IP analysis of interactions between GABARAPL2 and SIK2 deletion mutants (SIK2(1–290)-His, SIK2(1–550)-His, SIK2(1–650)-His, SIK2(1–931)-His) using anti-His antibodies. **n, o** Representative immunoblots and quantification of SIK2, p62, and LC3B in N2a-APP cells with SIK2 deletion mutants (*n* = 3/group). **p** Competitive binding. Purified full-length SIK2 protein were incubated with GST-GABARAPL2 and increasing concentrations of LIR peptide and subjected to GST pull-down. **q** Quantification of competitive binding in (**p**). **r** Volcano plot of differentially phosphorylated sites in N2a-APP-SIK2 versus N2a-APP cells. **s, t** Representative immunoblots and quantification of GABARAPL2, p62, and LC3B in N2a-APP cells with GABARAPL2 mutants (*n* = 3/group). **u** Representative immunoblots of GABARAPL2 and p-GABARAPL2(Ser72) in WT-control, WT-SIK2, 5 × FAD-control, and 5 × FAD-SIK2 mice (*n* = 6/group). **v** The quantification of p-GABARAPL2 in the dorsal hippocampus (*n* = 6/group). **w, x** N2a-APP cells overexpressing GABARAPL2-flag were transfected with control or SIK2 for 24 h. Immunoprecipitated with anti-flag antibody and probed with anti-p-Ser72-GABARAPL2 antibody by Western blot. Data are expressed as mean ± SEM. Statistical significance was calculated by unpaired two-tailed *t*-test (**x**), one-way ANOVA (**m, q**) and two-way ANOVA (**j, o, t, v**) followed by the Tukey’s post-hoc test. *P < 0.05, **P < 0.01, ***P < 0.001, ****P < 0.0001

To elucidate the molecular mechanisms underlying SIK2-mediated regulation of autophagic-lysosomal function, we conducted Co-IP assays coupled with MS in N2a cells overexpressing SIK2-flag. MS analysis identified members of the ATG8 family as potential SIK2 interactors (Fig. S7c). Subsequent Co-IP and Western blot validation confirmed that SIK2 physically associated with LC3A, LC3C, GABARAP, GABARAPL1, and GABARAPL2, but not LC3B (Fig. 5a–f). Reciprocal Co-IP experiments using flag-tagged ATG8 family members revealed the strongest interaction between SIK2 and GABARAPL2 (Fig. 5g, h). Hippocampal tissue Co-IP recapitulated SIK2-GABARAPL2 interaction (Fig. S7d). Importantly, this association exhibited strict isoform selectivity, as neither SIK1 nor SIK3 co-precipitated with GABARAPL2 in either experimental system (Fig. S7d). To determine whether GABARAPL2 is essential for SIK2-mediated autophagic regulation, we knocked down GABARAPL2 in SIK2-overexpressing N2a-APP cells. GABARAPL2 depletion significantly attenuated but did not fully abolish the SIK2-mediated p62 reduction (Fig. 5i, j), indicating GABARAPL2 is one of the key effector factors of SIK2 in autophagy modulation.

To map the SIK2-GABARAPL2 interaction domain, we generated a series of C-terminally truncated SIK2 constructs based on the LC3-interacting region (LIR motif: (W/F/Y)XX(L/I/V)) conserved in ATG8-binding partners (Fig. 5k), which engages the complementary LIR-docking site (LDS) on the surface of ATG8 family members [53]. Co-IP assays and truncation analysis revealed that the region spanning amino acids 290–550 mediates the interaction. Deletion of this region (ΔLIR-SIK2) abolished both the SIK2–GABARAPL2 interaction (Fig. 5l, m) and the subsequent reduction of p62 (Fig. 5n, o). Within 290–550, only one canonical LIR motif was identified at 326–333 residues. To confirm the necessity of this specific motif, we performed competitive GST pull-down assay. Purified full-length SIK2 protein was incubated with GST-GABARAPL2 in the presence of increasing concentrations (0, 5, 10, 20, 40 μmol/L) of a synthetic peptide spanning residues 326–333 (FAAIYFLL). The result showed that the LIR peptide competitively inhibited SIK2–GABARAPL2 binding in a dose-dependent manner, with a significant reduction at 20 and 40 μmol/L (Fig. 5p, q). A scrambled peptide (same amino acids, randomized sequence) showed no inhibition, confirming the specificity.

Next, we investigated whether SIK2 phosphorylates GABARAPL2 at serine or threonine residues. Immunoprecipitation of exogenetic GABARAPL2-flag from the SIK2-overexpressing N2a-APP cells followed by Western blotting with phospho-specific antibodies revealed phosphorylation at serine, but not threonine residues (Fig. S7e, f). Phosphoproteomic profiling identified three modifiable serine residues on GABARAPL2 (S39, S72, S87) in SIK2-overexpressing N2a-APP cells (Fig. 5r). To investigate the functional roles of these sites, we generated two types of GABARAPL2 mutant plasmids: phosphorylation-deficient mutants (S39A, S72A, S87A) and phosphomimetic mutants (S39E, S72E, S87E). Our results showed that expression of S72E alone was sufficient to reduce the expression of p62, whereas the other mutants unexpectedly caused an increase in p62 accumulation (Fig. 5s, t). None of the mutants affected total LC3B levels, confirming that S72 phosphorylation specifically enhances the autophagic flux.

The physiological significance of this mechanism was confirmed in vivo. We generated a phospho-specific antibody against GABARAPL2 Ser72. This antibody was validated by proportional signal loss upon GABARAPL2 knockdown (Fig. S7g, h), SIK2-dependent phosphorylation amplification (Fig. S7i, j), and abolished signal in S72A mutants but increased signal in S72E phospho-mimetics (Fig. S7k, l). Immunoblotting of hippocampal lysates from 5 × FAD-control mice showed reduced p-GABARAPL2 (ser72) levels compared to WT-control mice, which was reversed by SIK2 overexpression (Fig. 5u, v). To directly validate the SIK2-mediated phosphorylation of GABARAPL2 at Ser72, N2a-APP cells were co-transfected with either GABARAPL2-flag plus empty vector or GABARAPL2-flag plus SIK2 plasmid. Immunoprecipitation of GABARAPL2 using anti-flag antibody, and then Western blot probing with the p-GABARAPL2 (ser72) antibody (Fig. 5w) showed that SIK2 overexpression significantly increased p-GABARAPL2 (ser72) levels compared to vector control (Fig. 5w, x). The total GABARAPL2 level remained unchanged (Fig. 5w). Collectively, these findings demonstrate that SIK2 phosphorylates GABARAPL2 at Ser72 to enhance the autophagic flux.

### Phospho-GABARAPL2 at Ser72 alleviates the cognitive impairment in the middle-aged 5 × FAD mice

**Fig. 6:**
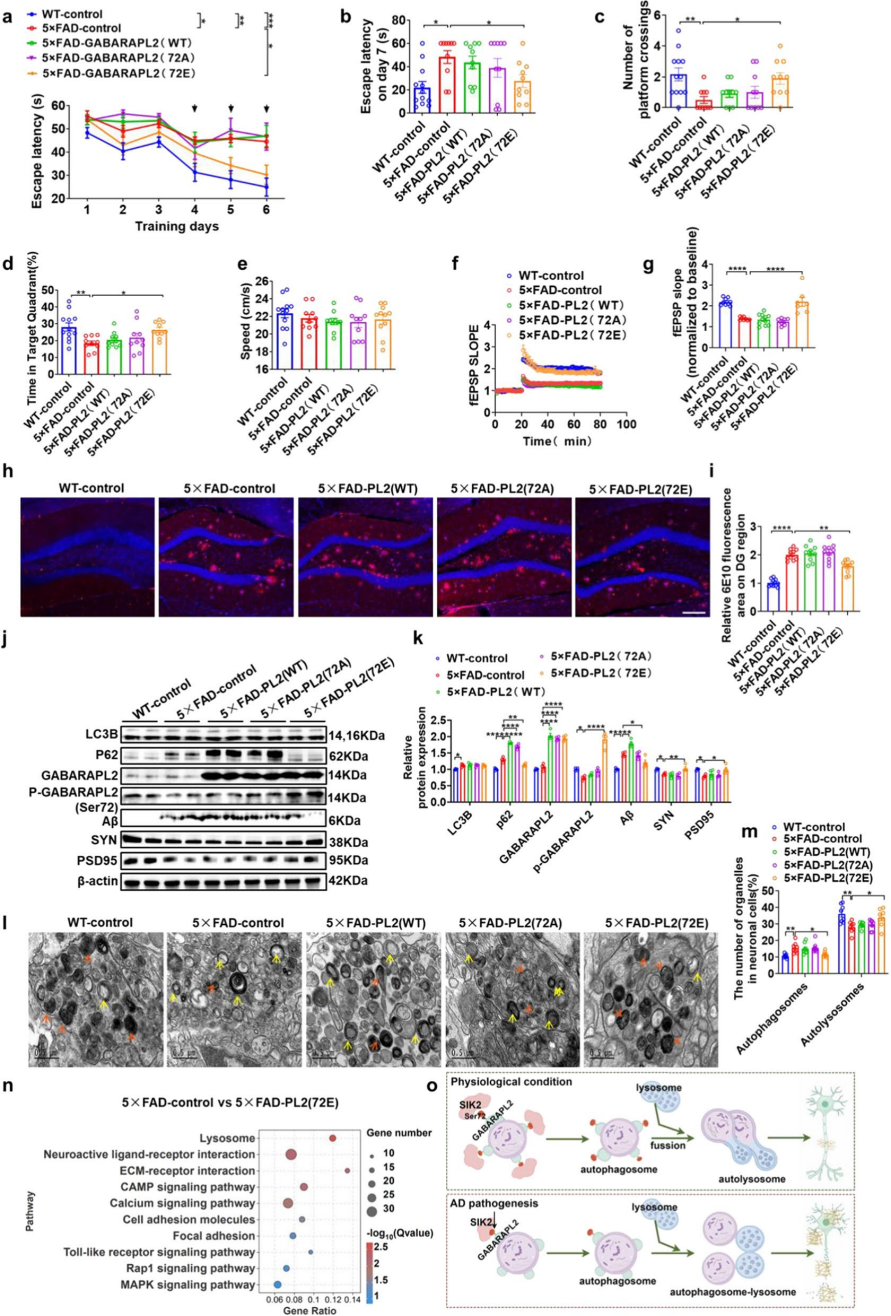
Phospho-GABARAPL2 at Ser72 alleviates the cognitive impairment of middle-aged 5 × FAD mice. **a-e** MWM performance of WT-control, 5 × FAD-control, 5 × FAD-PL2(WT), 5 × FAD-PL2(72A), and 5 × FAD-PL2(72E) mice. Escape latency during training trials (1–6 days) (**a**) and probe trial (day 7) (**b**). Platform crossings (**c**), percentage of time in the target quadrant (**d**), and swimming speed (**e**) during the probe trial (day 7). Sample sizes: *n* = 12 (WT-control), *n* = 10 (5 × FAD-control), *n* = 10 (5 × FAD-PL2(WT)), *n* = 10 (5 × FAD-PL2(72A)), *n* = 10 (5 × FAD-PL2(72E)). **f, g** Long-term potentiation (LTP) recordings in hippocampal CA1 regions. High-frequency stimulation (HFS) was applied, and fEPSP amplitudes were quantified during the last 10 min (**g**). Sample sizes: *n* = 3 mice, 7 slices per group. **h, i** Immunofluorescence staining for Aβ in the CA1 region. Representative images (**h**) and quantitative analysis (**i**). Scale bar, 50 µm. **j, k** Western blot analysis of GABARAPL2, p-GABARAPL2(Ser72), p62, LC3B, SYN, PSD95, and Aβ in dorsal hippocampal lysates. Representative immunoblots (**j**) and quantitative analyses (**k**) (*n* = 6 per group). **l, m** TEM images of autophagic vacuoles. Representative images (**l**) and quantitative analysis (**m**). Orange arrows: double-membrane autophagosomes; yellow arrows: single-membrane autolysosomes with electron-dense contents. Scale bars, 500 nm. **n** KEGG pathway analysis of DEGs between 5 × FAD-control and 5 × FAD-PL2(72E) mice, showing the top 10 enriched pathways. **o** Proposed model illustrating how SIK2 regulates autophagosome–lysosome fusion and neurodegeneration in AD. Data are expressed as mean ± SEM. Statistical significance was calculated by two-way ANOVA (**b, d-g, i, k, m**), and three-way ANOVA (**a**) followed by the Tukey’s post-hoc test, and Scheirer-Ray-Hare test followed by the Dunn’s post-hot test (**c**). *P < 0.05, **P < 0.01, ***P < 0.001, ****P < 0.0001

To explore the role of GABARAPL2 phosphorylation in AD pathogenesis, neuron-specific AAVs expressing *GABARAPL2* variants were stereotaxically injected in the hippocampus of 6-month-old 5 × FAD mice and WT littermates. In the MWM, the 5 × FAD-control mice showed longer escape latencies during training trials (days 4–6) and the probe trial (day 7) compared to the WT-control mice. Notably, the escape latencies were significantly reduced in 5 × FAD-GABARAPL2(S72E) mice on day 6 of training and in the probe trial (day 7) compared to the 5 × FAD-control mice (Fig. 6a, b). GABARAPL2(S72E) overexpression also significantly increased the platform crossings (Fig. 6c) and the time spent in the target quadrant (Fig. 6d) in the probe test compared to the 5 × FAD-control mice. No significant differences in the swimming speed were observed among the groups (Fig. 6e). Electrophysiological recordings revealed impaired LTP maintenance in the 5 × FAD-control mice, which was rescued by GABARAPL2(S72E) overexpression (Fig. 6f, g). Western blotting revealed reduced SYN and PSD95 levels in the 5 × FAD-control mice versus the WT-control mice. Overexpressing GABARAPL2(S72E) restored SYN and PSD95 levels in 5 × FAD mice (Fig. 6j, k). These results collectively indicate that upregulation of GABARAPL2(S72E) restores synaptic plasticity and alleviates cognitive impairment in middle-aged 5 × FAD mice.

Immunofluorescence analysis showed significantly higher Aβ plaque deposition in the hippocampus of 5 × FAD-control mice compared to the WT-controls (Fig. 6h, i). Remarkably, the 5 × FAD-GABARAPL2(S72E) mice had markedly reduced Aβ plaque burden compared to the 5 × FAD-controls, whereas GABARAPL2-WT or GABARAPL2-S72A overexpression did not induce significant changes (Fig. 6h, i). Western blot quantification of Aβ_42_ levels confirmed these findings, showing significant reductions in 5 × FAD-GABARAPL2(S72E) mice relative to 5 × FAD-controls (Fig. 6j, k). The total GABARAPL2 protein level increased comparably across all overexpression groups. The level of p-GABARAPL2 (ser72) was reduced in 5 × FAD-control compared to WT-control mice but significantly elevated only in the GABARAPL2(S72E) overexpression group. P62 accumulation in the 5 × FAD-control versus WT-control mice was exacerbated in the 5 × FAD-GABARAPL2-WT and -S72A mice but reduced in the 5 × FAD-GABARAPL2-S72E group. LC3B levels remained elevated in the 5 × FAD-control mice and unchanged across intervention groups (Fig. 6j, k).

We further performed transcriptomic profiling in hippocampal tissues from 5 × FAD-GABARAPL2(S72E) and 5 × FAD-control mice. PCA analysis demonstrated a clear separation between the two groups, indicating that GABARAPL2(S72E) overexpression induces distinct transcriptomic patterns (Fig. S6d). Consistently, RNA-seq analysis identified 344 upregulated and 338 downregulated genes (FDR < 0.05) in the 5 × FAD-GABARAPL2(S72E) mice compared to the 5 × FAD-controls (Fig. S6e), with lysosomal pathways being the most enriched (Fig. 6n). TEM demonstrated a significant increase in double-membrane autophagosomes in 5 × FAD-control mice compared to WT-controls. This defect was substantially rescued in 5 × FAD-GABARAPL2(S72E) mice, which showed reduced autophagosome accumulation and increased mature autolysosomes (Fig. 6l, m). Collectively, these data demonstrate that the phosphomimetic S72E mutation enhances the ability of GABARAPL2 to rescue the autophagic flux, mitigate Aβ pathology, and improve cognitive deficits in 5 × FAD mice.

## Discussion

In this study, we provide compelling evidence that SIK2 dysfunction exacerbates AD-related pathology through impaired autophagic flux and compromised synaptic plasticity. We observed a progressive decline in SIK2 expression in 5 × FAD mice and AD patients, which correlated with spatial memory deficits and Aβ accumulation. Notably, up-regulation of SIK2 can promote the formation of autophagic vacuoles and the maturation of autophagic lysosomes by phosphorylating GABARAPL2, reduce Aβ plaque burden, restore synaptic ultrastructure, and rescue the cognitive impairment in middle-aged 5 × FAD mice (Fig. 6o). These findings align with prior studies linking autolysosome deficits to AD progression [14, 20, 54–56], but extend this paradigm by identifying SIK2 as a critical upstream regulator of autophagosome–lysosome fusion.

Pioneering proteomic analyses of the autophagy interaction network in human cells have identified several serine/threonine kinases, including AMPK, PI3K, TBK1, STK3, STK4, and NEK9, as part of the ATG8s interactome [30]. The major autophagy-regulating protein kinases ULK1/2 bind to ATG8s via LIR motifs [54, 55]. To determine whether SIK2 interacts with GABARAPL2 in a LIR-dependent manner, Co-IP assays using C-terminally truncated SIK2 constructs revealed that the interaction is mediated by the C-terminal LIR region. The competitive GST pull-down assay further confirmed the necessity of this specific motif. Previous studies have established that TBK1 phosphorylates GABARAPL2 at Ser10, Ser39, Ser87 and Ser88 [34]. Our phosphoproteomics revealed three residues—Ser39, Ser72 and Ser87—whose phosphorylation is strictly SIK2-dependent. Among the three sites, Ser72 phosphorylation had not been reported previously. Site-directed mutagenesis confirmed that the phospho-mimetic mutation of Ser72 alone was sufficient to rescue autophagosome–lysosome fusion, whereas equivalent mutations at Ser39 or Ser87 were ineffective. These findings indicate that the SIK2-mediated phosphorylation of Ser72 promotes autophagosome–lysosome fusion, thereby distinguishing SIK2 from TBK1, whose primary role remains confined to mitophagy. This mechanism could also explain the rescue of Aβ pathology in SIK2-overexpressing 5 × FAD mice, as enhanced autophagic flux accelerates Aβ clearance. Importantly, the phosphomimetic mutant GABARAPL2-S72E replicated the cognitive benefits of SIK2 overexpression, underscoring the functional significance of this post-translational modification.

Emerging evidence highlights the isoform-specific roles of SIKs in AD pathogenesis. SIK1 and SIK3 have distinct signaling axes, substrate preferences, and spatiotemporal regulation of Aβ deposition and neuroinflammatory cascades [45, 56]. Our supplementary data showed that SIK2 overexpression did not alter SIK1 or SIK3 level in 5 × FAD mice (Fig. S4g, h), indicating that the observed effects on autophagy, Aβ clearance, and synaptic plasticity are specifically mediated by SIK2 rather than other SIK isoforms. This finding aligns with the evidence of functional divergence among SIK family members and underscores the need to target SIK2 specifically for AD therapy.

SIK2 also plays a role in synaptic plasticity. Its upregulation restored LTP and dendritic spine density in 5 × FAD mice, accompanied by increased levels of synaptic markers (PSD-95, SYN) and BDNF. These effects may result from both reduced Aβ toxicity via enhanced autophagy and decreased neuroinflammation. Our data suggest that neuronal SIK2 overexpression reduces neuroinflammation in 5 × FAD mice. Western blot analysis revealed elevated microglial and astrocyte activation in 5 × FAD-control mice, indicated by increased ionized calcium binding adaptor molecule 1 (Iba1) and glial fibrillary acidic protein (GFAP) levels, compared to WT-controls. SIK2 overexpression significantly reduced Iba1 and GFAP expression in 5 × FAD mice versus the 5 × FAD-controls, while the WT-SIK2 mice showed reduced Iba1 levels relative to WT-controls (Fig. S4g, h). Immunofluorescence confirmed diminished GFAP⁺ and Iba1⁺ signals in 5 × FAD-SIK2 mice compared to 5 × FAD-controls (Fig. S4i–l). Conversely, SIK2 knockdown increased Iba1 and GFAP expression in 5 × FAD-shSIK2 and WT-shSIK2 mice compared to their respective controls (Fig. S5c–f). This anti-inflammatory effect likely stems from SIK2-induced enhancement of Aβ clearance, reducing plaque-driven glial activation and modulating inflammatory signaling.

SIK2 is emerging as a pleiotropic kinase integrating metabolic and immune signaling, with its dysregulation potentially driving multiple pathological cascades. In immune regulation, SIK2 maintains homeostasis by controlling lymphocyte maturation [57] and mediating IL-33-dependent cytokine/chemokine secretion in mast cells [58], while its deficiency exacerbates immune injury and inflammation. Mechanistically, SIK2 modulates the metabolic-inflammatory crosstalk via distinct pathways: it suppresses hepatic gluconeogenesis and alleviates lipogenesis-driven inflammation through the CRTC2-ACC1 axis [59], and coordinates HDAC4 phosphorylation to regulate NF-κB signaling [60]. SIK2 activity is dynamically regulated by the microenvironment, being downregulated by TNFα in adipocytes [61] and modulated by endoplasmic reticulum stress via POMC secretome homeostasis [62]. Additionally, the functions of SIK2 extend to fibrosis regulation in lung injury [63] and selenoprotein hierarchy in macrophage redox balance [64]. These findings position SIK2 as a key regulator of metabolic reprogramming, immune modulation, and neuroendocrine signaling, suggesting its potential role in AD-associated neuroinflammation through multi-target coordination. Our study shows that SIK2 is predominantly expressed in neurons, with lower levels in microglia throughout the central nervous system; however, its full mechanistic contribution to microglial pathophysiology remains to be explored. Overall, SIK2 acts as a multi-faceted modulator of AD pathology, autophagic-lysosomal function, synaptic integrity, and neuroinflammation.

Despite robust findings, study limitations should be considered. First, the study primarily used the 5 × FAD model, which predominantly mimics amyloid pathology. Validating these results in tauopathy models (e.g., 3 × Tg-AD) would enhance translational relevance. Second, the therapeutic potential of SIK2 activators in middle-aged AD mice remains untested due to the lack of commercially available specific SIK2 activators.

## Conclusions

In conclusion, this work establishes SIK2 as a pivotal regulator of autophagy and synaptic function in AD. Age-dependent SIK2 loss disrupts proteostasis, worsening Aβ deposition and cognitive decline. Targeting the SIK2–GABARAPL2 axis may provide a dual therapeutic strategy to enhance Aβ clearance and preserve synaptic integrity in AD.

## Supplementary Information

Additional file 1. **Table S1.** List of antibodies utilized in experiments.** Figure S1**. Autophagic flux impairment in 5×FAD mice. **Figure S2**. Expression patterns of SIK2 in the mouse brain during development. **Figure S3**. The effect of SIK2 knockdown on cognitive function of 5×FAD mice. **Figure S4**. The effect of SIK2 knockdown on gliosis in 5×FAD mice. **Figure S5**. The effect of SIK2 overexpression on the synaptic plasticity and gliosis in 5×FAD mice. **Figure S6**. Phosphoproteomic and transcriptomic profiling reveals gene regulation by SIK2-mediated phosphorylation of GABARAPL2-S72E in AD models. **Figure S7**. Multimodal characterization of SIK2-GABARAPL2 interaction and phosphoregulation.

Additional file 2. All original, full-length gel and blot images.

## Data Availability

The authors declare that all data supporting the findings of this study are available in this article and its Supplementary information files. Further inquiries can be directed to the corresponding author.

## Abbreviations

AAV: Adeno-associated virus
Aβ: Amyloid-β
AD: Alzheimer’s disease
APP: Amyloid precursor protein
AMPK: AMP-activated protein kinase
BDNF: Brain-derived neurotrophic factor
DEGs: Differentially expressed genes
ELISA: Enzyme-linked immunosorbent assay
FDR: False discovery rate
fEPSPs: Field excitatory postsynaptic potentials
GABARAPL2: GABA type A receptor-associated protein-like 2
GFAP: Glial Fibrillary acidic protein
EGFP: Enhanced green fluorescent protein
Iba1: Ionized calcium binding adaptor molecule 1
IF: Immunofluorescence assays
Co-IP: Co-immunoprecipitation
GST: Glutathione S-transferase
KEGG: Kyoto encyclopedia of genes and genomes
LTP: Long-term potentiation
MWM: Morris water maze
MS: Mass spectrometry
PSD: Postsynaptic density
PSD95: Postsynaptic density protein 95
RNA-seq: RNA sequencing
qPCR: Quantitative polymerase chain reaction
SC: Synaptic cleft
SIK2: Salt-inducible kinase 2
SYN: Synaptophysin
TEM: Transmission electron microscopy
PCA: Principal component analysis
