# Supplementary material for: SIK2-mediated phosphorylation of GABARAPL2 facilitates autophagosome–lysosome fusion and rescues neurodegeneration in an Alzheimer’s disease model
Source: Transl Neurodegener. 2025 Oct 23;14:53. doi: 10.1186/s40035-025-00514-4 (PMC12548284; doi:10.1186/s40035-025-00514-4)
Supplement: Supplementary file 1 — Additional file 1. Table S1. List of antibodies utilized in experiments. Figure S1. Autophagic flux impairment in 5×FAD mice. Figure S2. Expression patterns of SIK2 in the mouse brain during development. Figure S3. The effect of SIK2 knockdown on cognitive function of 5×FAD mice. Figure S4. The effect of SIK2 knockdown on gliosis in 5×FAD mice. Figure S5. The effect of SIK2 overexpression on the synaptic plasticity and gliosis in 5×FAD mice. Figure S6. Phosphoproteomic and transcriptomic profiling reveals gene regulation by SIK2-mediated phosphorylation of GABARAPL2-S72E in AD models. Figure S7. Multimodal characterization of SIK2-GABARAPL2 interaction and phosphoregulation. [file 40035_2025_514_MOESM1_ESM.docx]

**Supplemental Information**

Supplementary Table S1

Supplementary Figures S1-7

**SIK2-Mediated Phosphorylation of GABARAPL2 Facilitates Autophagosome-Lysosome Fusion and Rescues Neurodegeneration in an Alzheimer’s Disease Model**

Xiaoman Dai^1,2^†, Ziling Ye^2^†, Chen Wang^1^†, Yufei Huang^2^, Yun Chen^2^, Tianqing Han^2^, Weijie Gao^2^, Xin Wu^2^, Jing Zhang^1,2^*, Xiaochun Chen^1,2^*

**Affiliations**

^1^ Department of Neurology and Geriatrics, Fujian Institute of Geriatrics, Fujian Medical University Union Hospital, 29 Xinquan Road, Fuzhou, Fujian 350001, China.

^2^ Fujian Key Laboratory of Molecular Neurology and Institute of Neuroscience, School of Basic Medical Sciences, Fujian Medical University, 88 Jiaotong Road, Fuzhou, Fujian 350001, China.

| **Antibodies** | **Company** | **Reference** | **Use** |
| --- | --- | --- | --- |
| GAPDH | Abcam | ab9485 | WB,1:5000 |
| β-actin | Abcam | ab8226 | WB,1:5000 |
| APP | Millipore | 17160 | WB,1:1000 |
| BACE1 | Abcam | Ab2077 | WB,1:1000 |
| Aβ (6E10) | Biolegend | 803001 | WB,1:1000 |
| GFAP | SYSY | 173 011 | WB,1:1000 |
| Iba1 | Abcam | ab 15690 | WB,1:1000 |
| NeuN | Abcam | ab104224 | WB,1:1000 |
| SIK1 | Abcam | Ab217809 | WB,1:1000 |
| SIK2 | Cell Signaling | 6919 | WB,1:1000 |
| SIK3 | Abcam | ab88495 | WB,1:1000 |
| PSD95 | Abcam | ab18258 | WB,1:1000 |
| Synaptophysin | Cell Signaling | 9020S | WB,1:1000 |
| BDNF | Abcam | ab108319 | WB,1:1000 |
| LAMP1 | Abcam | ab25245 | WB,1:1000 |
| LC3B | Sigma | L7543 | WB,1:1000 |
| LC3A | Cell Signaling | 4599 | WB,1:1000 |
| LC3B | Cell Signaling | 3868 | WB,1:1000 |
| LC3C | Cell Signaling | 14736 | WB,1:1000 |
| GABARAP | Cell Signaling | 13733 | WB,1:1000 |
| GABARAPL1 | Cell Signaling | 26632 | WB,1:1000 |
| GABARAPL2 | Cell Signaling | 14256 | WB,1:1000 |
| Phospho-GABARAPL2(Ser72) | Jingjie PTM BioLab (Hangzhou) Co. Ltd | Customized | WB,1:500 |
| Phosphoserine | Boster | BM1622 | WB,1:1000 |
| Phosphothreonine | Abcam | Ab218195 | WB,1:1000 |
| Phospho-HDAC4 | Cell Signaling | 3443 | WB,1:1000 |
| GST | Thermo Fisher Scientific | MA4-004 | WB,1:2000 |
| Flag | Sigma | F7425 | IP：4-5 µl |
| IgG | Cell Signaling | 2729P | IP:2 µl |
| His | Proteintech | 66005-1-Ig | IP：5 µl， WB,1:1000 |
| SIK2 | Affinity | DF10140 | IF,1:200 |
| GFAP | Abcam | ab254083 | IF:1:500 |
| Iba1 | Wako | 019-19741 | IF:1:500 |
| NeuN | Cell Signaling | ab104224 | IF:1:500, WB,1:1000 |
| MAP2 | Millipore | MAB 3418 | IF,1:100 |

**Table S1. List of antibodies utilized in experiments.**

WB, western blot; IP, Co- immunoprecipitation; IF, immunofluorescence assays.


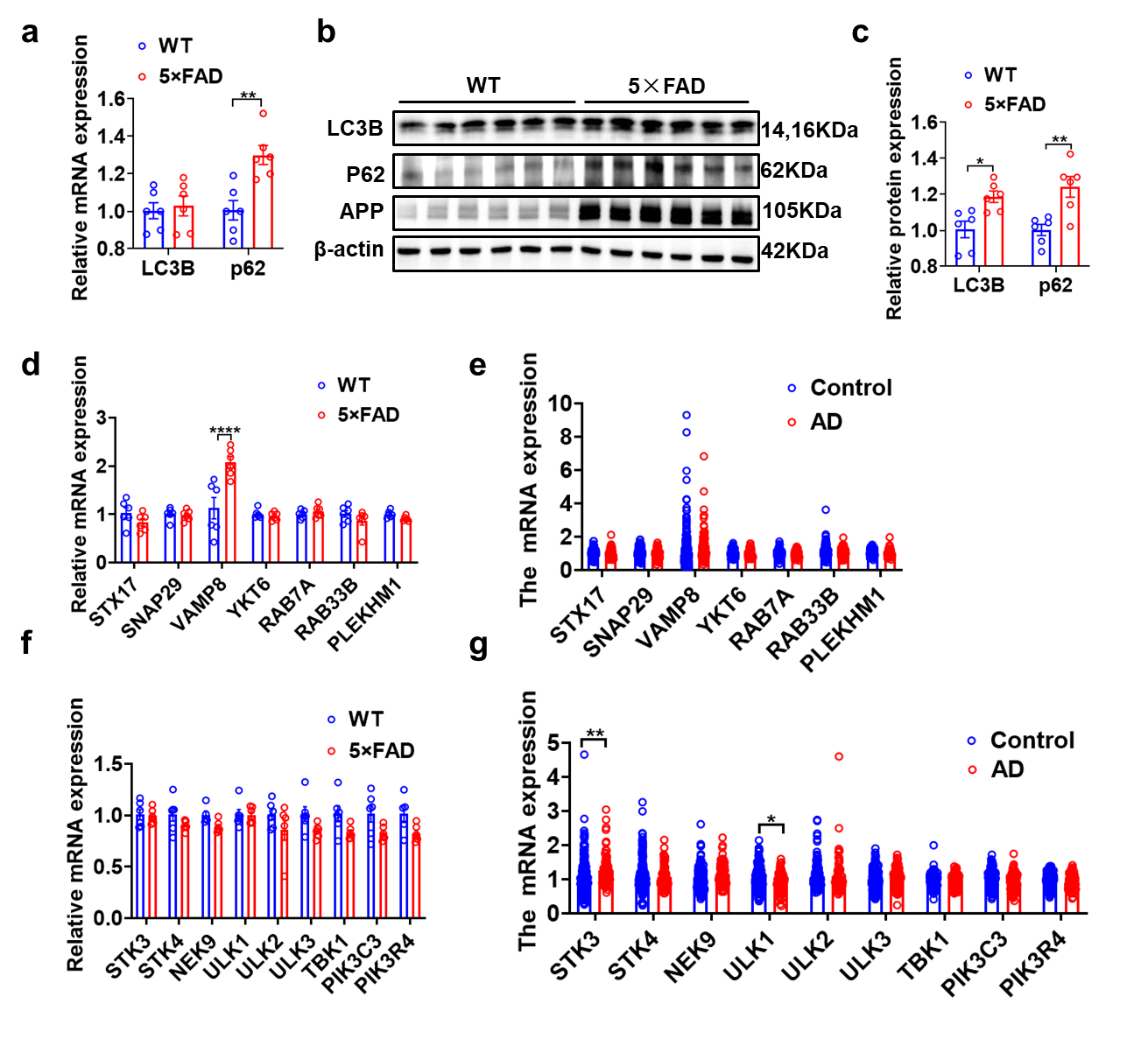


**Figure S1. Autophagic flux impairment in 5×FAD mice.**

**a** qPCR analysis of LC3B and p62 mRNA levels in the hippocampus of 10-month-old WT and 5×FAD mice (*n* = 6/group). **b-c** Western blot analysis of LC3B and p62 protein levels in the hippocampus of 10-month-old WT and 5×FAD mice (*n* = 6/group). **d** qPCR analysis of autophagosome trafficking-related mRNA levels in the hippocampus of 10-month-old WT and 5×FAD mice (*n* = 6/group). **e** Bioinformatics analysis of autophagosome trafficking-related gene expression in the temporal cortex of AD patients compared to controls. **f** qPCR analysis of autophagosome-associated kinase mRNA levels in the hippocampus of 10-month-old WT and 5×FAD mice (*n* = 6/group). **g** Bioinformatics analysis of autophagosome-associated kinase gene expression in the temporal cortex of AD patients compared to controls. Data are expressed as mean ± SEM. Statistical signiicance was calculated by two-way ANOVA (**a,c,d-g**) followed by the Tukey’s post-test. **P* < 0.05, ***P* < 0.01, *****P* < 0.0001.


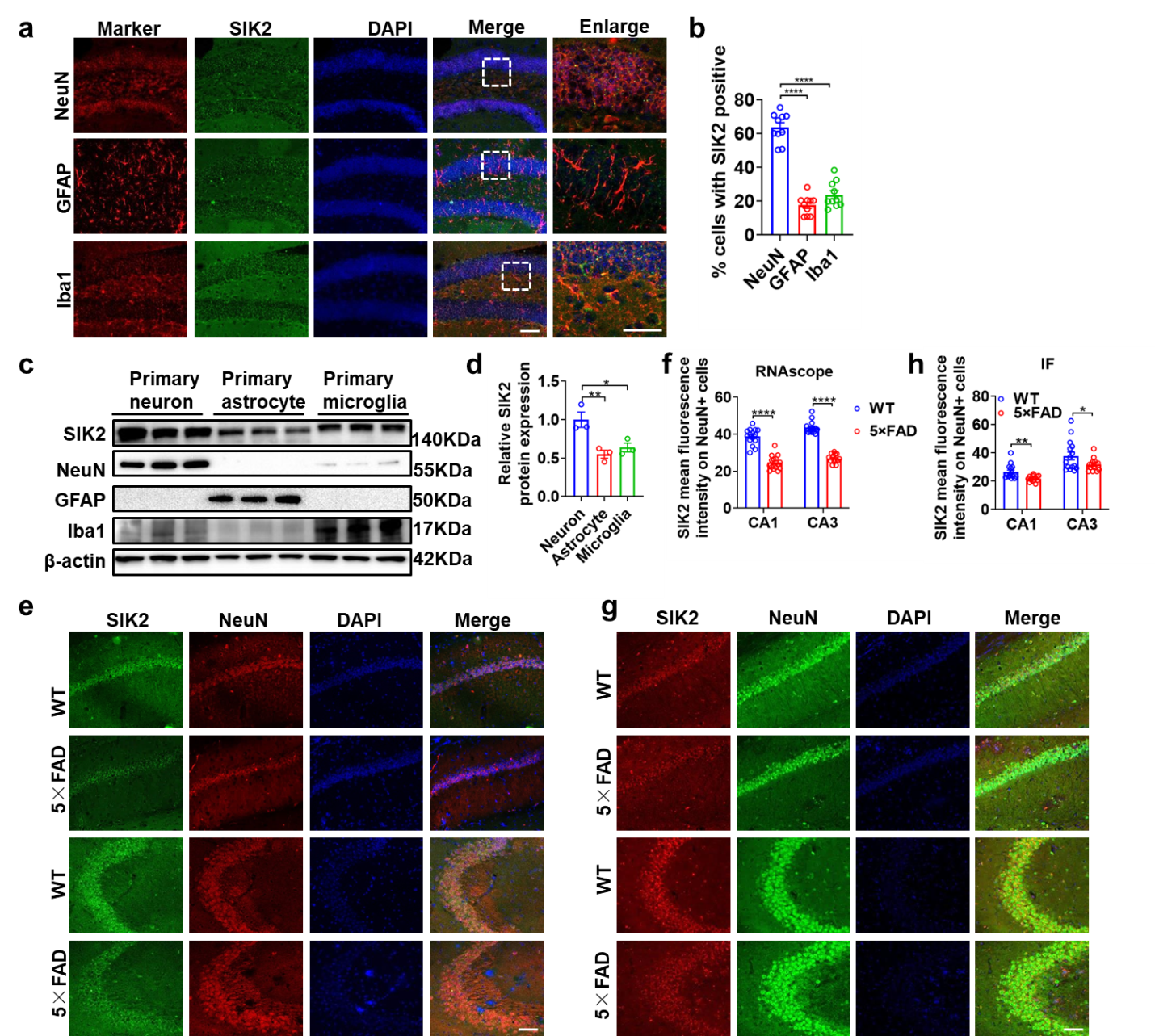


**Figure S2. Expression patterns of SIK2 in the mouse brain during development.**

**a-b** Double-labeled confocal immunofluorescence images showing colocalization of SIK2 (green) with the neuronal marker NeuN (red, top), astrocyte marker GFAP (red, middle), or microglia marker Iba1 (red, bottom) in the dentate gyrus (DG) of the mouse hippocampus (*n* = 3). Scale bar = 50 µm. **c-d** Representative immunoblots and quantification of SIK2 in primary neurons, astrocytes, and microglia (*n* = 3). **e-f** SIK2-specific RNA probes combined with NeuN immunofluorescence showing colocalization of SIK2 (green) and NeuN (red) in the CA1 (top) and CA3 (bottom) regions of WT and 5×FAD mice. Scale bar = 50 µm. Quantification of SIK2 intensity in CA1 and CA3 NeuN⁺ cells (*n* = 3/group). **g-h** Double-labeled immunofluorescence showing colocalization of SIK2 (red) and NeuN (green) in the CA1 (top) and CA3 (bottom) regions of WT and 5×FAD mice. Scale bar = 50 µm. Quantification of SIK2 intensity in CA1 and CA3 NeuN⁺ cells (*n* = 3/group). Data are expressed as mean ± SEM. Statistical significance was calculated by unpaired two-tailed t-test (**f,h**) and one-way ANOVA (**b,d**) followed by the Tukey’s post-test. **P* < 0.05, ***P* < 0.01, ****P* < 0.001, *****P* < 0.0001.

**
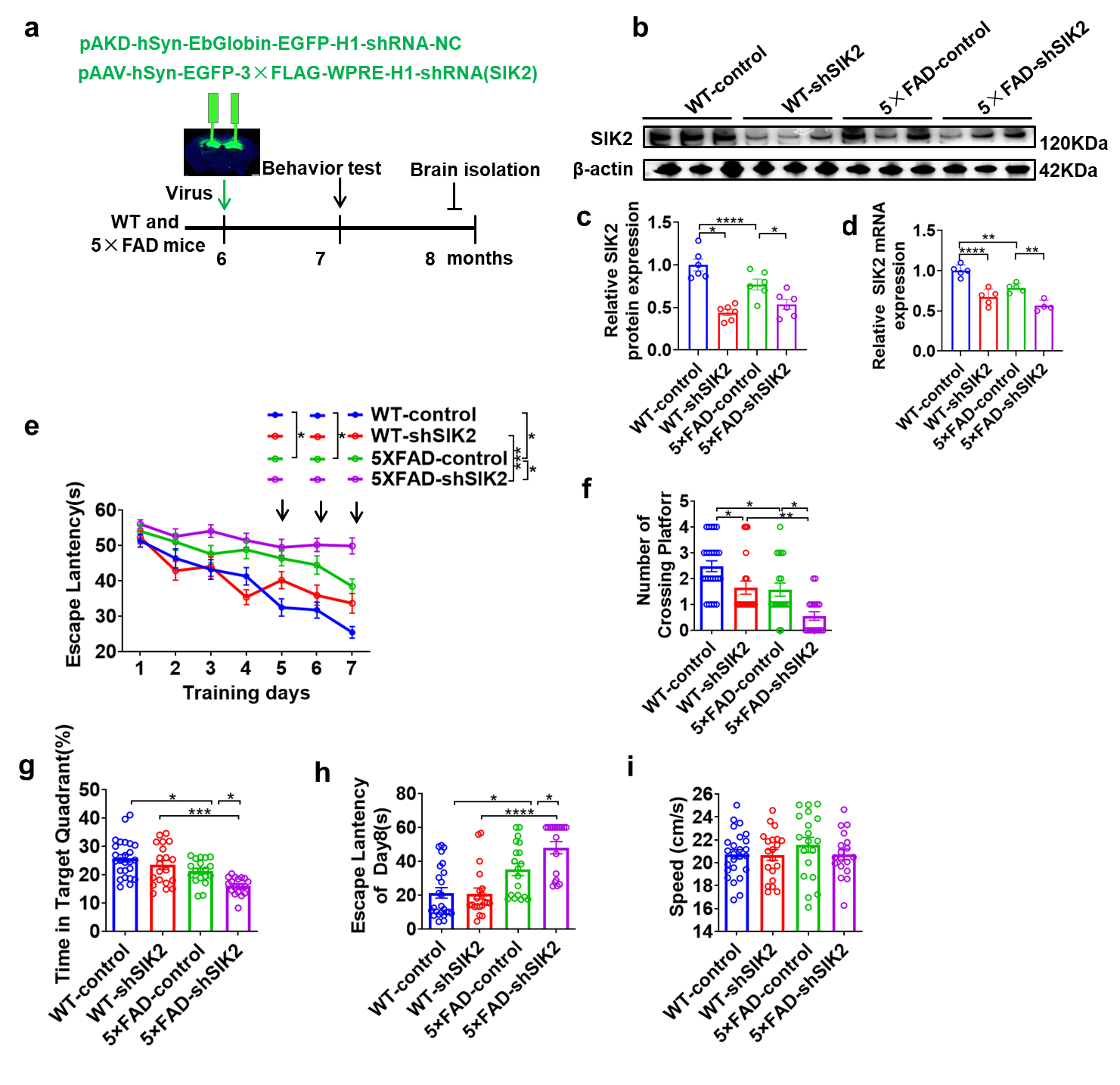
**

**Figure S3. The effect of SIK2** **knockdown on cognitive function of 5×FAD mice.**

**a** Experimental timeline for WT and 5×FAD mice. **b-c** Representative immunoblots and quantification of SIK2 in the dorsal hippocampus of WT-control, WT-shSIK2, 5×FAD-control, and 5×FAD-shSIK2 mice (*n* = 6/group). **d** Knockdown efficiency of SIK2 assessed by qPCR (*n* = 5). **e-i** Morris water maze (MWM) performance of WT-control, WT-shSIK2, 5×FAD-control, and 5×FAD-shSIK2 mice. Escape latency during training trials (1-7 days) (**e**) and probe trial (day 8) (**h**). Platform crossings (**f**), percentage of time in the target quadrant (**g**), and swimming speed (**i**) during the probe trial (day 8). Sample sizes: *n* = 25 (WT-control), *n* = 20 (WT-shSIK2), *n* = 19 (5×FAD-control), *n* = 18 (5×FAD-shSIK2). Data are expressed as mean ± SEM. Statistical significance was calculated by two-way ANOVA (**c-d,f-i**) and three-way ANOVA (**e**) followed by the Tukey’s post-test, and Scheirer-Ray-Hare test followed by the Dunn’s post-hot test (**f**). **P* < 0.05, ***P* < 0.01, ****P* < 0.001, *****P* < 0.0001.

**
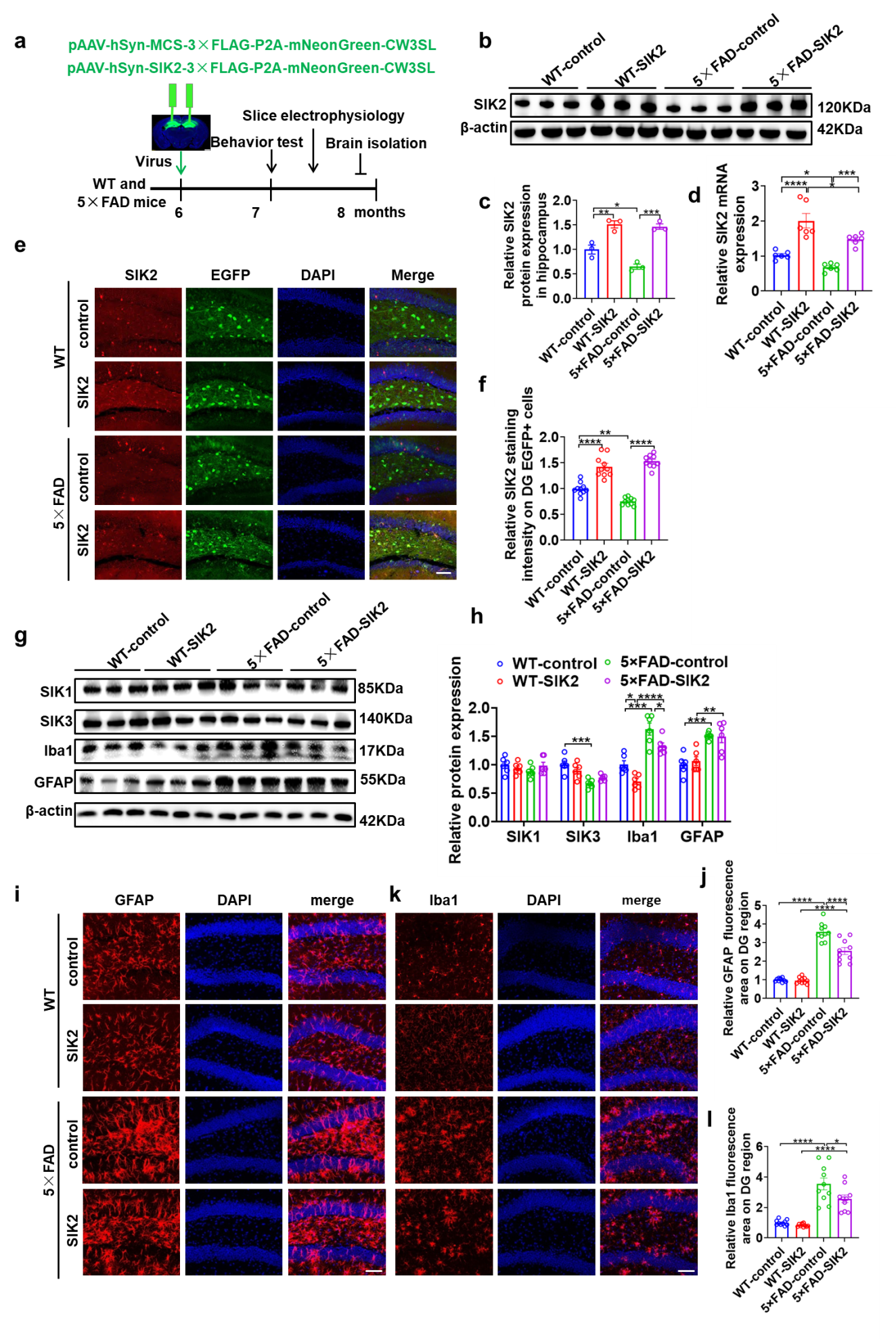
**

**Figure S4. The effect of SIK2 knockdown on gliosis in 5×FAD mice.**

**a** Experimental timeline for WT and 5×FAD mice. **b-c** Representative immunoblots and quantification of SIK2 in the dorsal hippocampus of WT-control, WT-SIK2, 5×FAD-control, and 5×FAD-SIK2 mice (*n* = 3/group). **d** Overexpression efficiency of SIK2 assessed by qPCR (*n* = 6). **e-f** Overexpression efficiency of SIK2 assessed by immunofluorescence (*n* = 3/group). Scale bar = 50 µm. **g-h** Representative immunoblots and quantification of SIK1, SIK3, GFAP, and Iba1 in the dorsal hippocampus of WT-control, WT-SIK2, 5×FAD-control, and 5×FAD-SIK2 mice (*n* = 6/group). **i-l** Immunofluorescence staining for GFAP and Iba1 in the DG region. Representative images (**i, k**) and quantification (**j, l**) (*n* = 3/group). Scale bar = 50 µm. Data are expressed as mean ± SEM. Statistical significance was calculated by two-way ANOVA(**c-d,f,h,j**) followed by the Tukey’s post-test. **P* < 0.05, ** *P* < 0.01, *** *P* < 0.001, **** *P* < 0.0001.


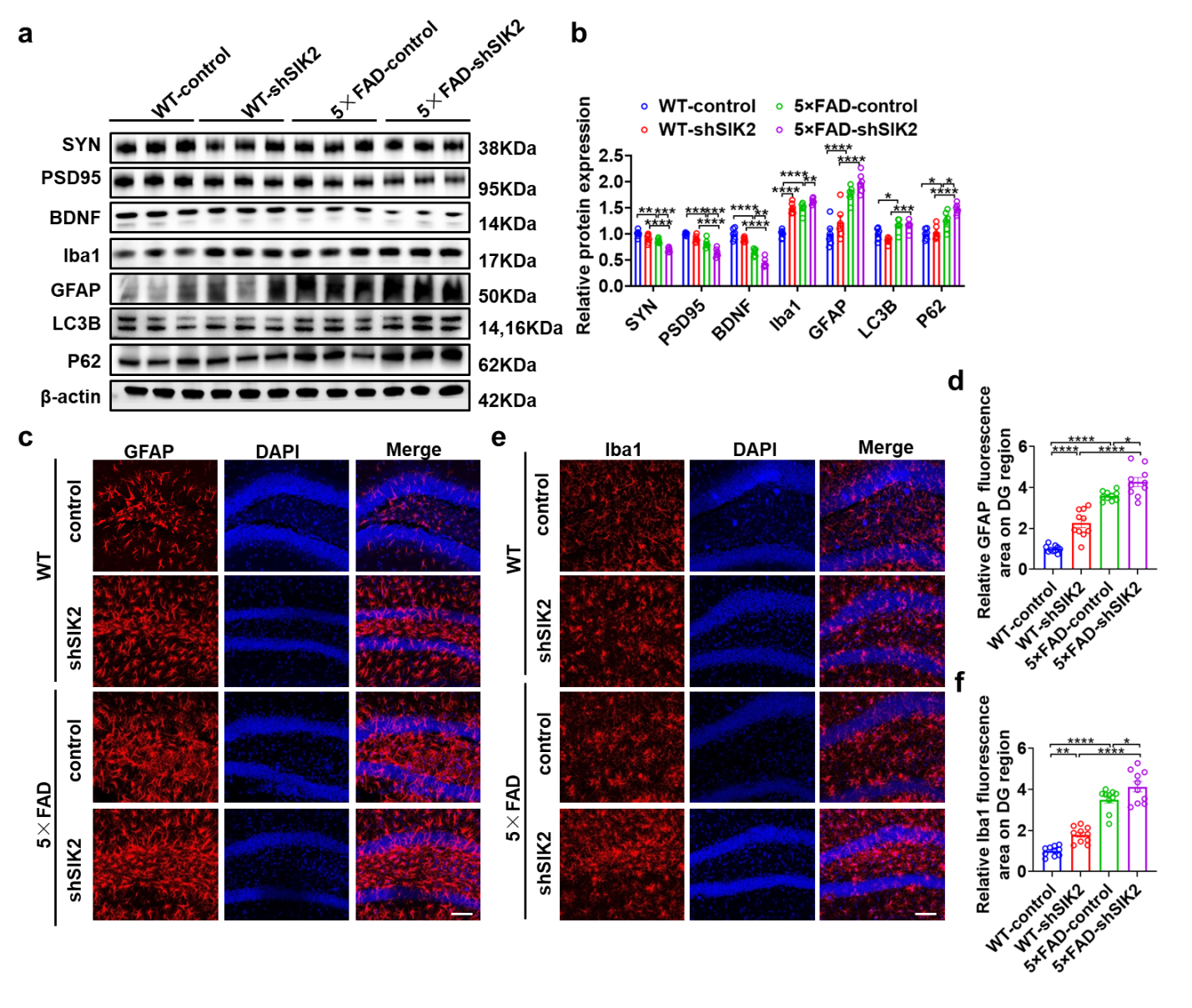


**Figure S5. The effect of SIK2 overexpression on the synaptic plasticity and gliosis in 5×FAD mice.**

**a-b** Representative immunoblots and quantification of SYN, PSD95, BDNF, GFAP, Iba1, LC3B, and p62 in the dorsal hippocampus (*n* = 6/group). **c-d** Representative immunofluorescence images and quantification of GFAP staining in the DG region (*n* = 3/group). Scale bar = 50 µm. **e-f** Representative immunofluorescence images and quantification of Iba1 staining in the DG region (*n* = 3/group). Scale bar = 50 µm. Data are expressed as mean ± SEM. Statistical significance was calculated by two-way ANOVA(**b,d,f,h**) followed by the Tukey’s post-test. **P* < 0.05, ** *P* < 0.01, *** *P* < 0.001, **** *P* < 0.0001.

**
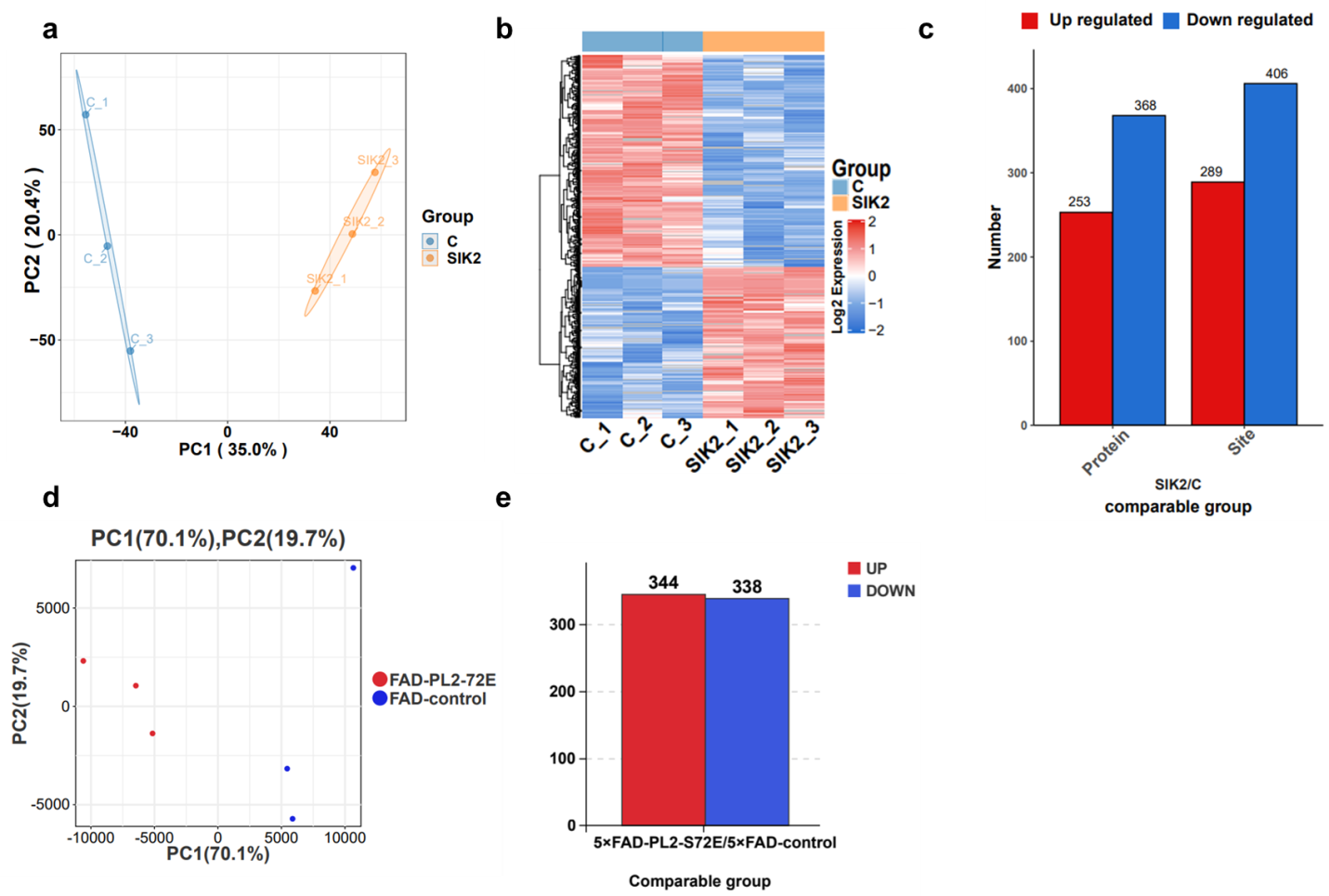
**

**Figure S6.** **Phosphoproteomic and transcriptomic profiling reveals gene regulation by SIK2-mediated phosphorylation of GABARAPL2-S72E in AD models.**

**a** Principal component analysis (PCA) of the phosphoproteome in N2a-APP-SIK2 cells (orange) and N2a-APP-control cells (blue). **b** Heatmap showing distinct protein expression patterns in N2a-APP-SIK2 versus N2a-APP cells (fold change >1.5, *P* < 0.05). **c** Summary of identified differentially expressed proteins and phosphorylated sites. **d** Principal component analysis (PCA) of hippocampal gene expression profiles in 5×FAD-control (blue) and 5×FAD-PL2-72E (red) mice. **e** Bar graph showing upregulated (344 genes, red) and downregulated (338 genes, blue) transcripts in 5×FAD-GABARAPL2-S72E hippocampus compared to 5×FAD-controls (fold change >1.2, *P* < 0.05).


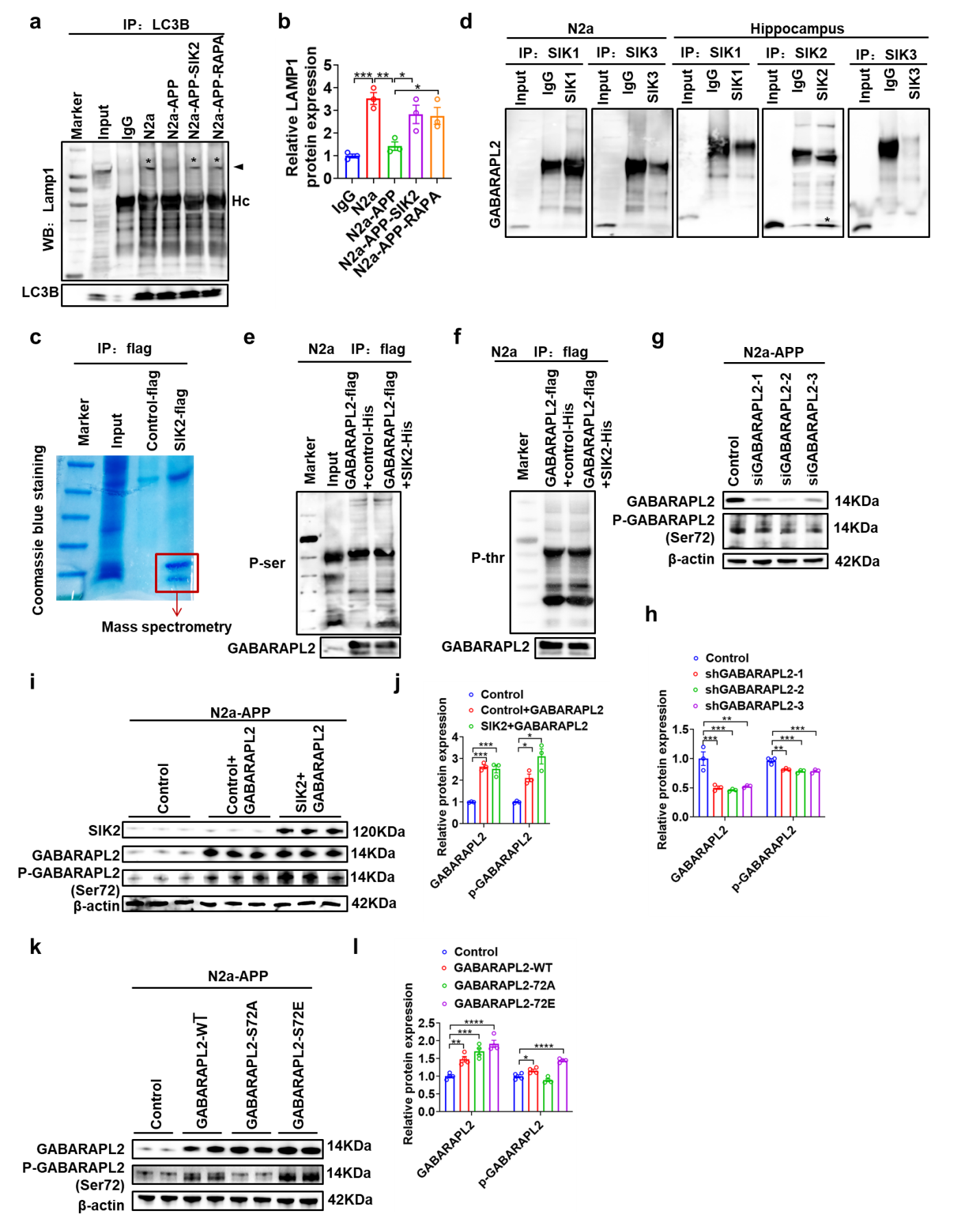


**Figure S7.** **Multimodal characterization of SIK2-GABARAPL2 interaction and phosphoregulation.**

**a** Co-IP analysis of the LC3B-LAMP1 interaction using anti-LC3B antibody in N2a, N2a-APP, N2a-APP-SIK2, and N2a-APP-RAPA (rapamycin-treated) cells. Asterisks indicate co-precipitated LAMP1 (120 kDa). Hc: IgG heavy chain. **b** The quantification of LAMP1 in the cell groups (*n* = 3/group). **c** Coomassie blue-stained SDS-PAGE gel of anti-flag immunoprecipitates from N2a cells expressing control-flag (lane 3) or SIK2-flag (lane 4). Red boxes indicate SIK2-specific interacting proteins. **d** Co-IP analysis of interactions between SIK1, SIK2, SIK3 and GABARAPL2 in N2a cells and hippocampal tissues. **e-f** N2a-APP cells overexpressing GABARAPL2-flag were transfected with control or SIK2 for 1 day. Immunoprecipitated with anti-flag antibody, and probed with anti-phospho-Ser (**e**) and anti-phospho-Thr antibodies (**f**) by Western blot. **g-h** The representative immunoblotting and quantification of GABARAPL2 and p-GABARAPL2 (ser72) after siGABARAPL2-1, siGABARAPL2-2, and siGABARAPL2-3 siRNAs were transferred into N2a-APP cells (*n* = 3/group). **i-j** The representative immunoblotting and quantification of SIK2, GABARAPL2 and p-GABARAPL2 (ser72) in N2a-APP-Control, N2a-APP -GABARAPL2, N2a-APP-SIK2-GABARAPL2 cells (*n* = 3/group). **k-l** The representative immunoblotting and quantification of GABARAPL2 and p-GABARAPL2 (ser72) in the groups (*n* = 3/group). Data are expressed as mean ± SEM. Statistical significance was calculated by two-way ANOVA(**b, h, j, l**) followed by the Tukey’s post-test. **P* < 0.05, ** *P* < 0.01, *** *P* < 0.001, **** *P* < 0.0001.
