# Supplementary figures and images for: SIK2-mediated phosphorylation of GABARAPL2 facilitates autophagosome–lysosome fusion and rescues neurodegeneration in an Alzheimer’s disease model
Source: Transl Neurodegener. 2025 Oct 23;14:53. doi: 10.1186/s40035-025-00514-4 (PMC12548284; doi:10.1186/s40035-025-00514-4)

**Fig1.**

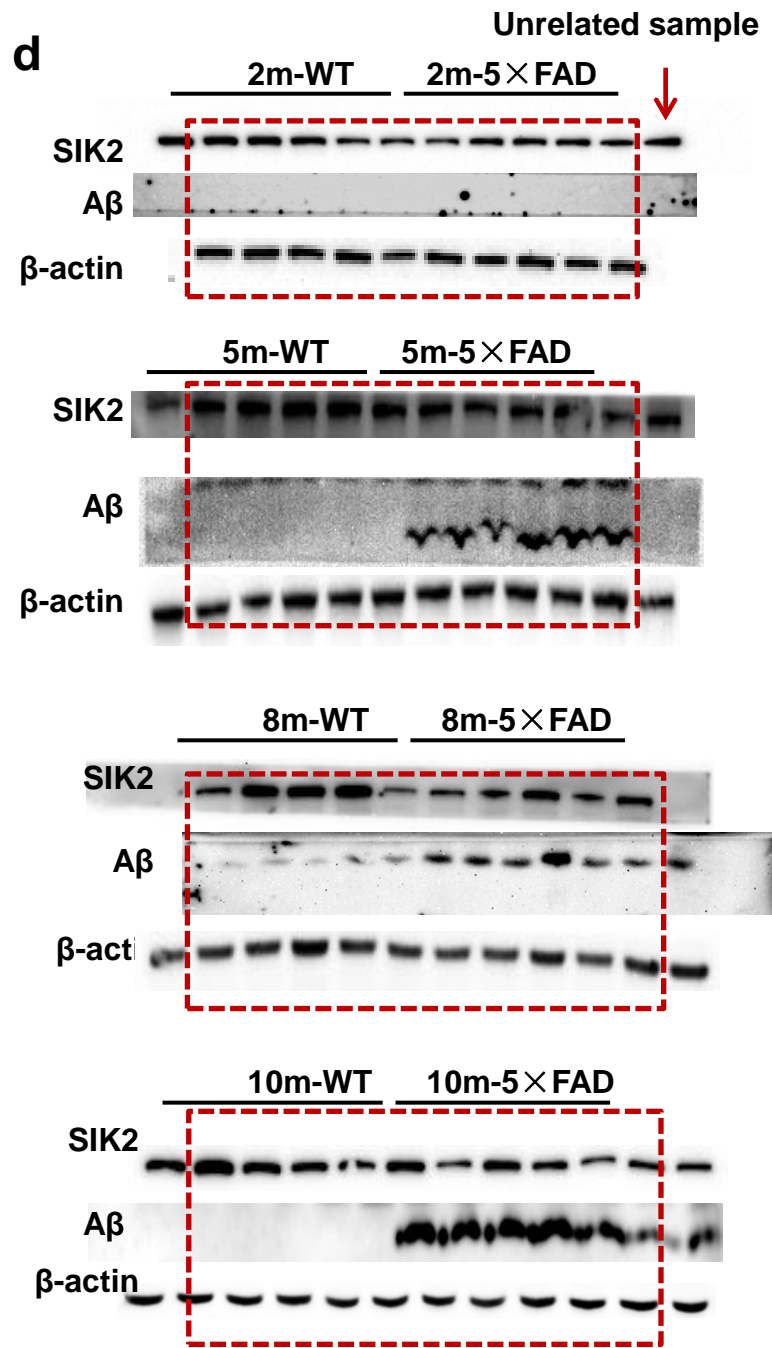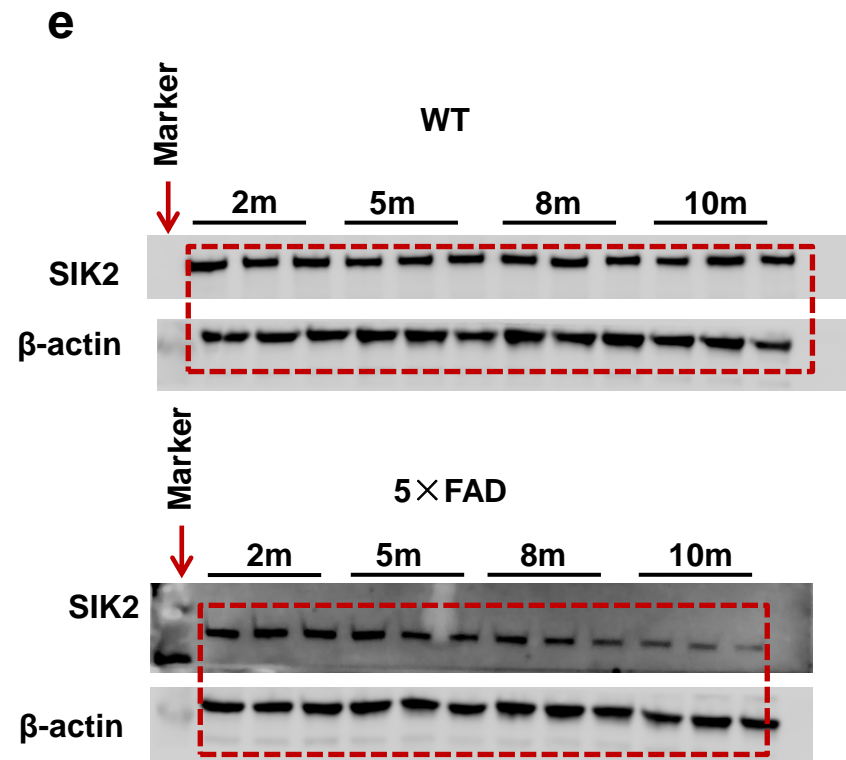

Fig2.

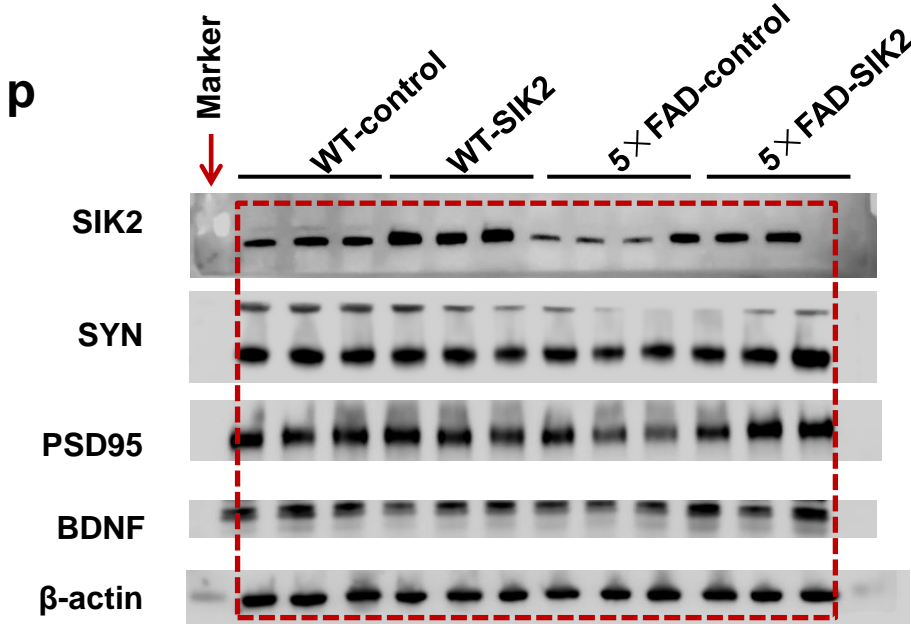

Fig3.

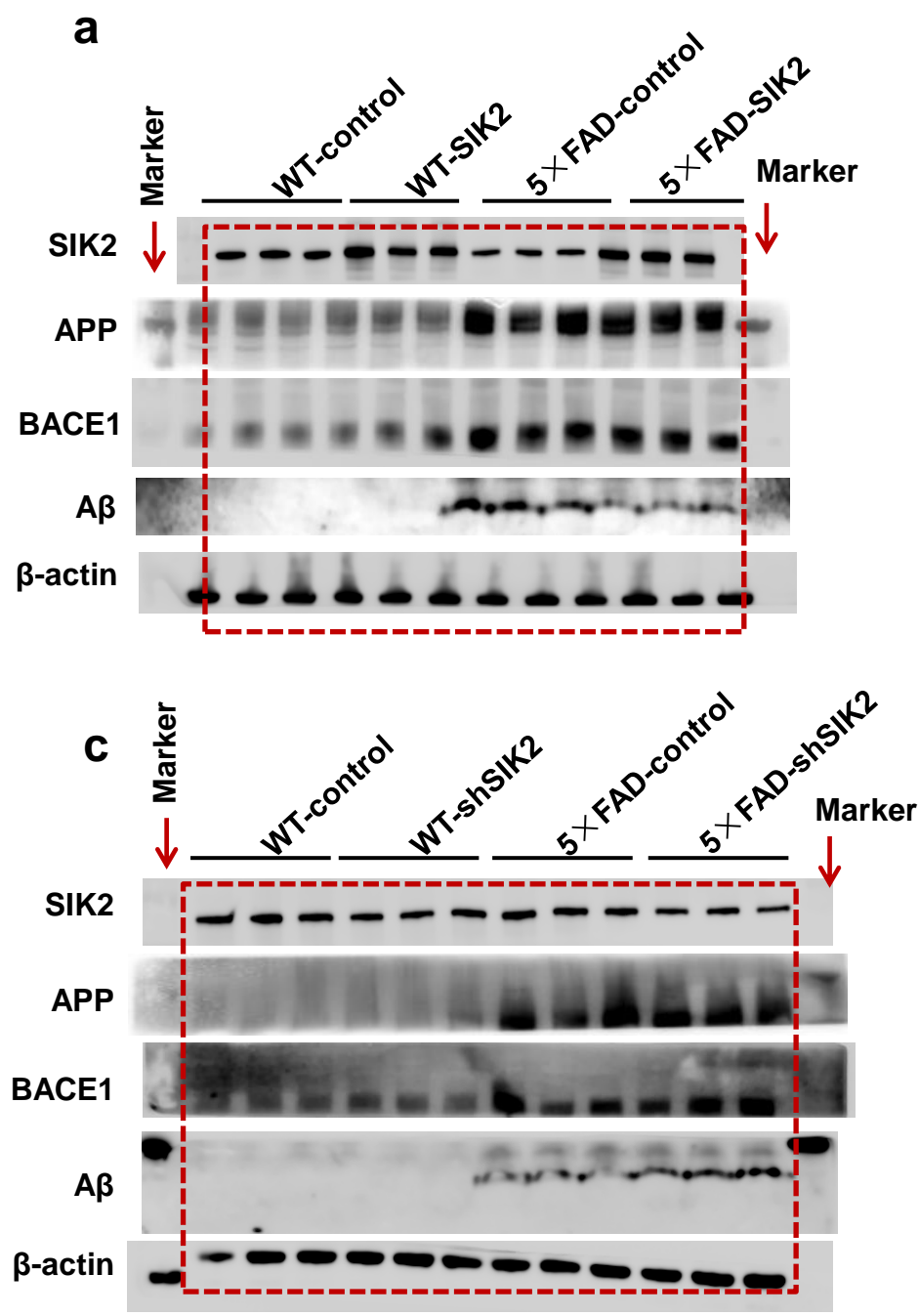

**Fig4.**

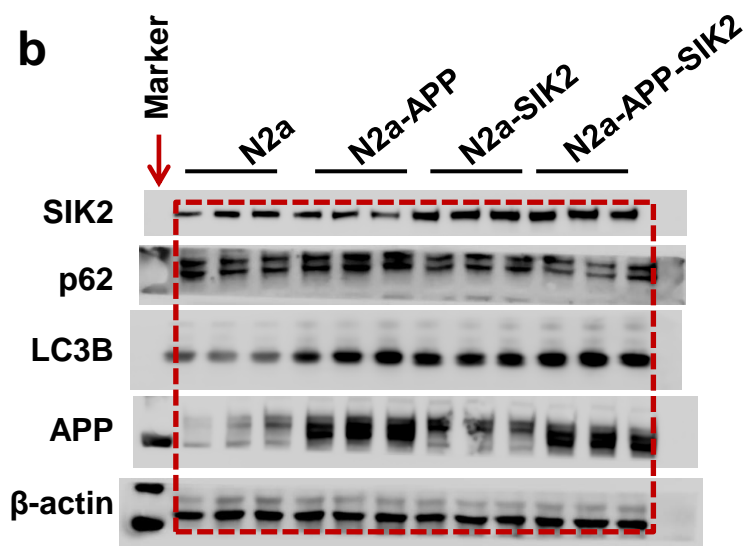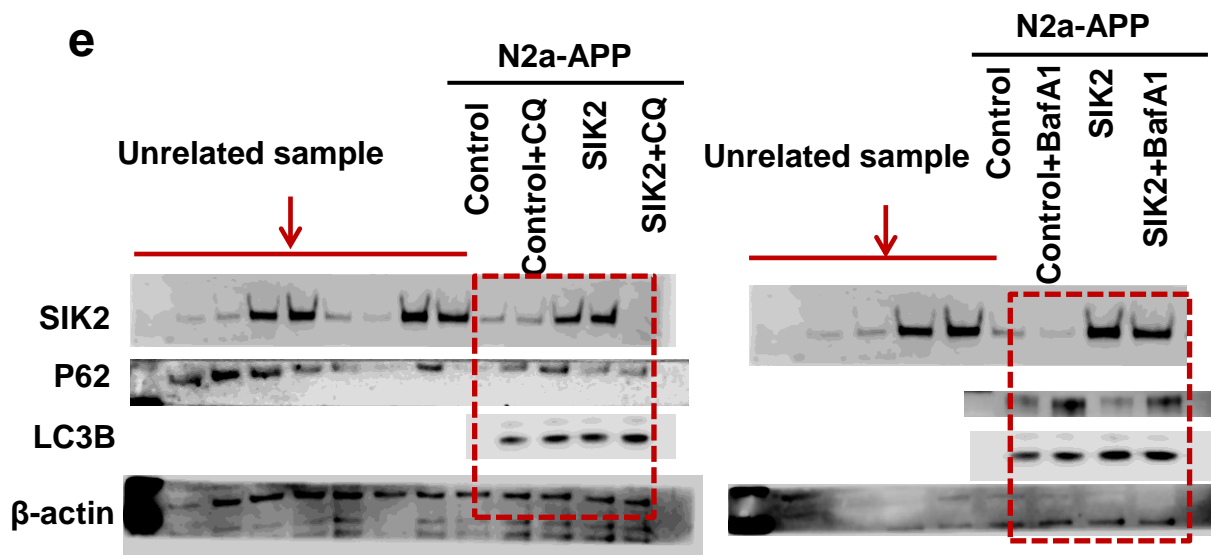

**Fig4.**

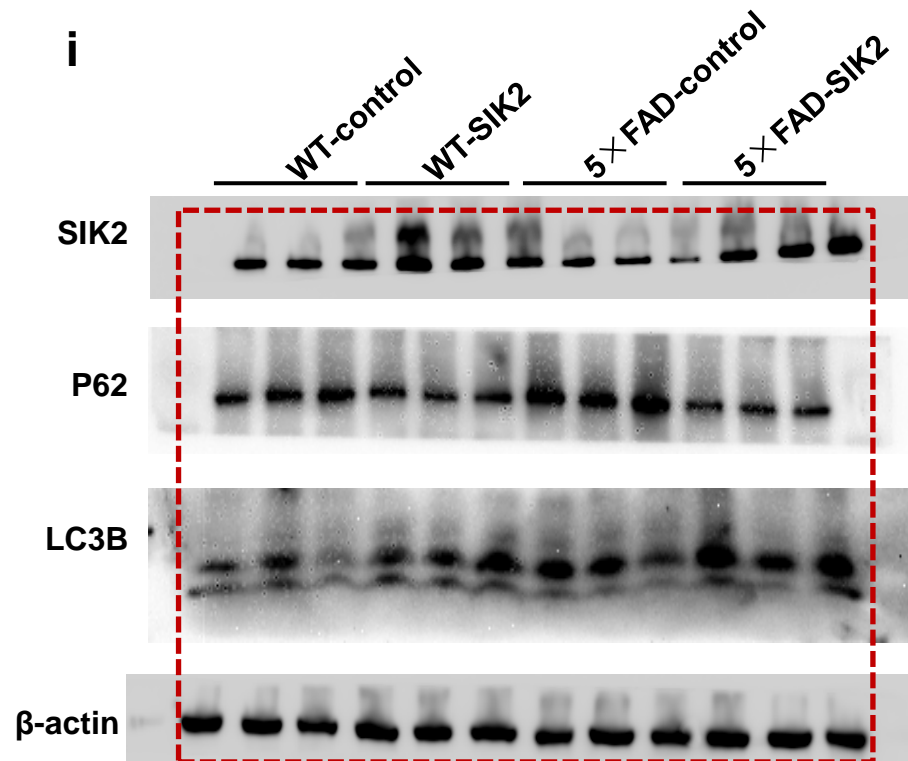

Fig5.

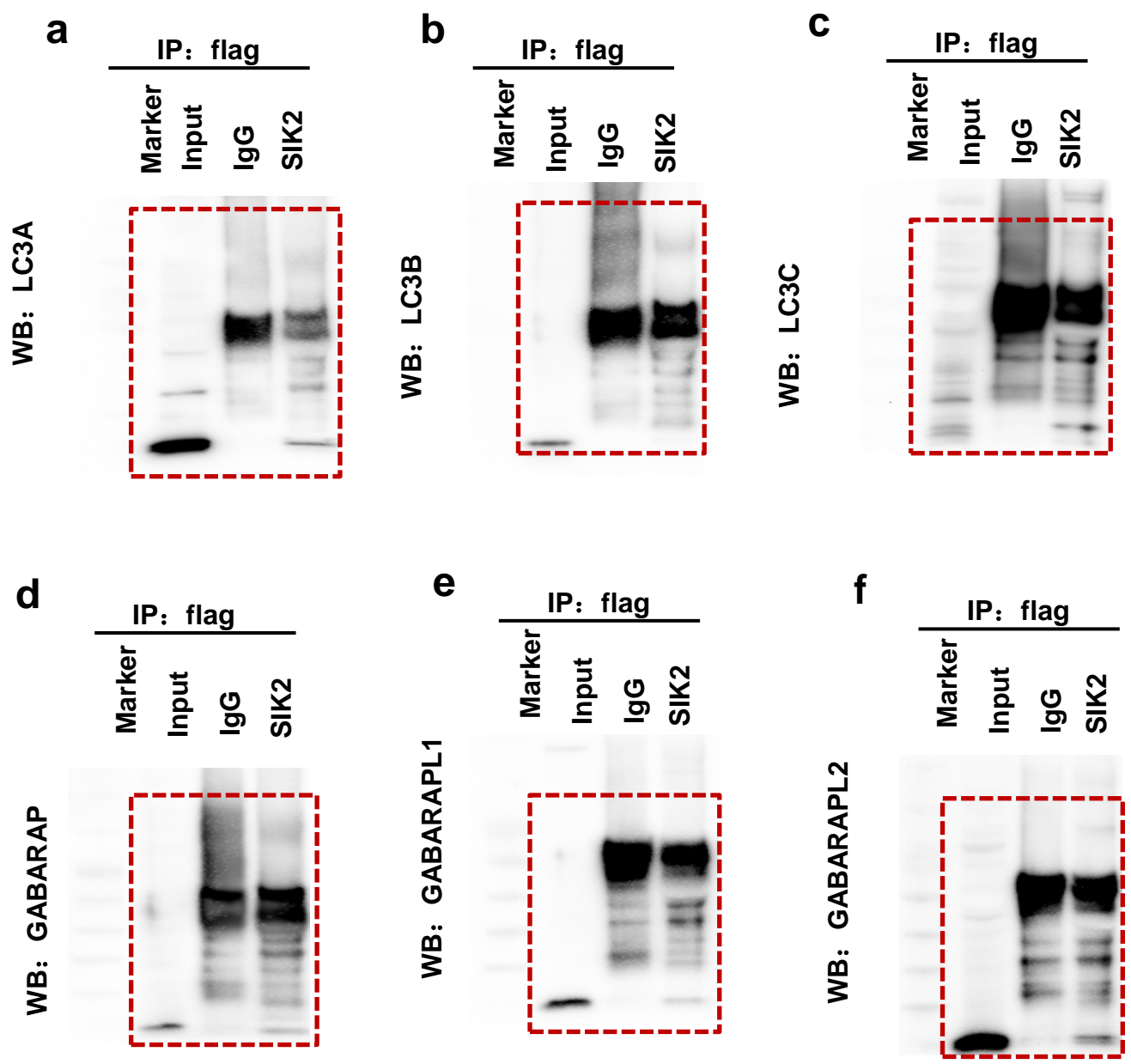

**Fig5. g**

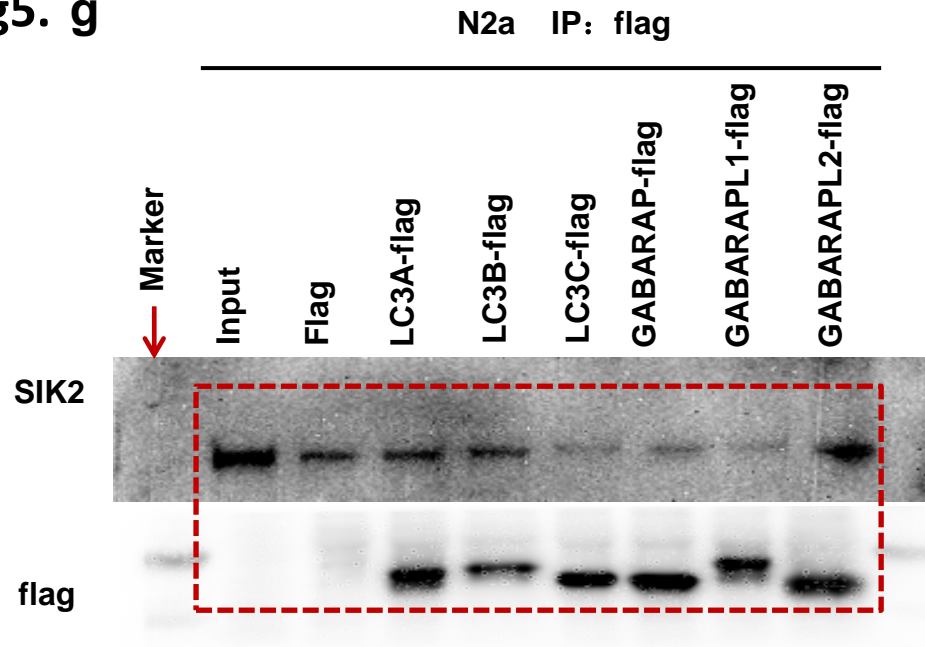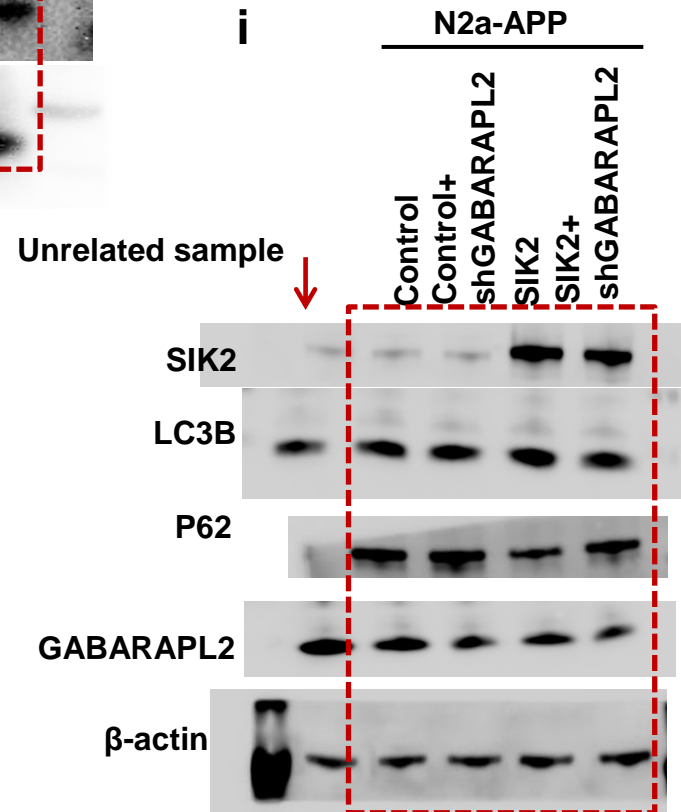

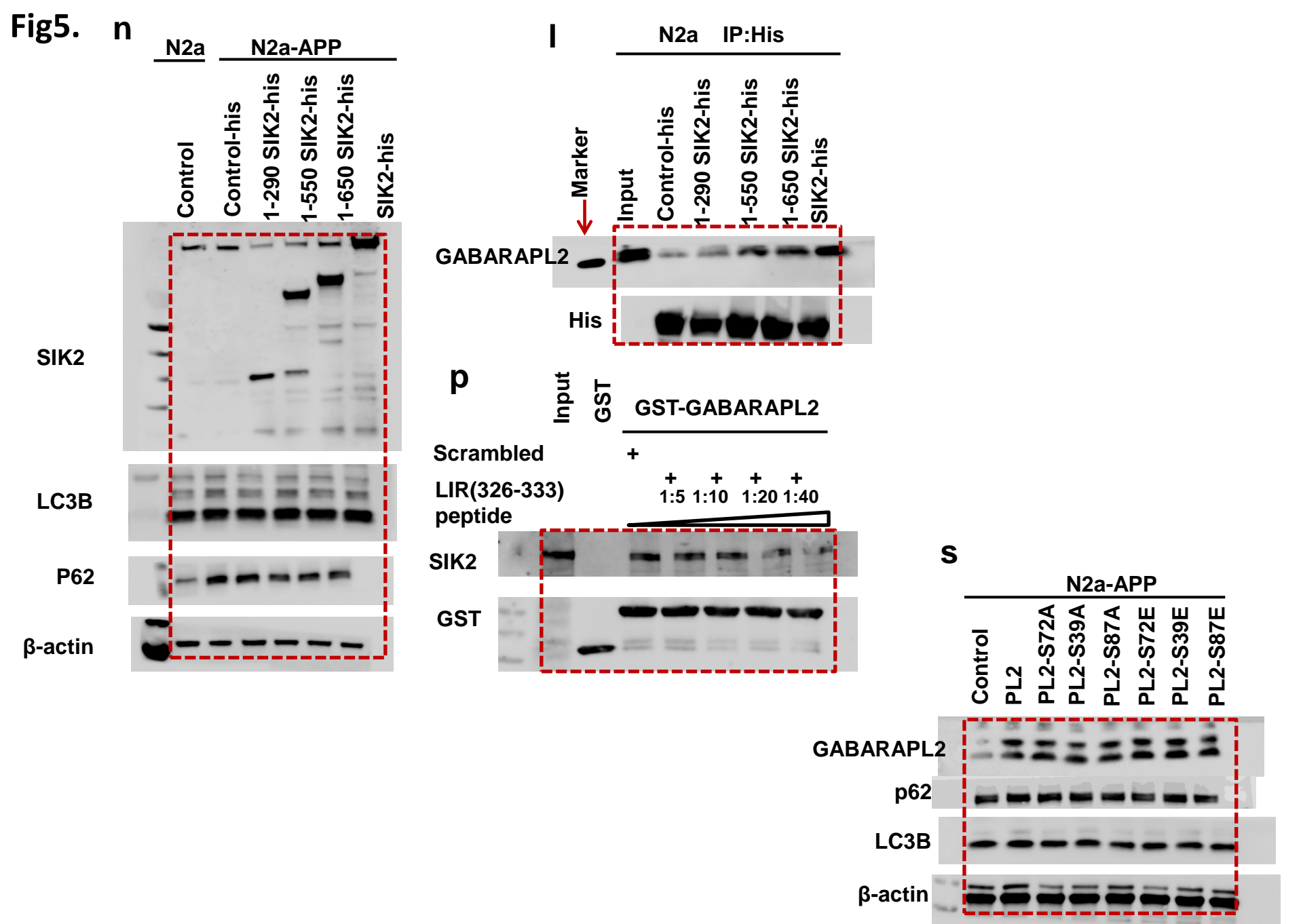

**p**

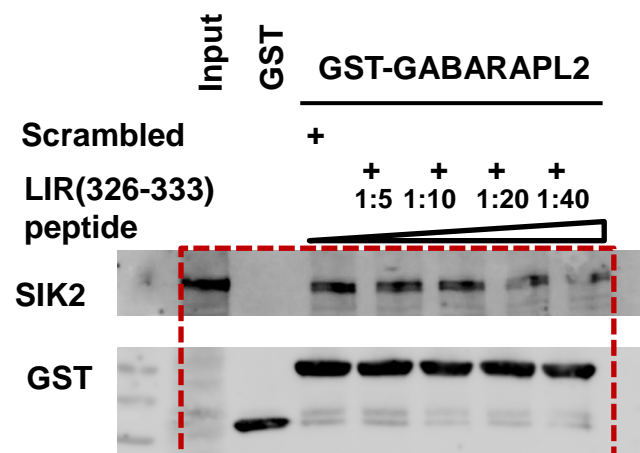

**u**

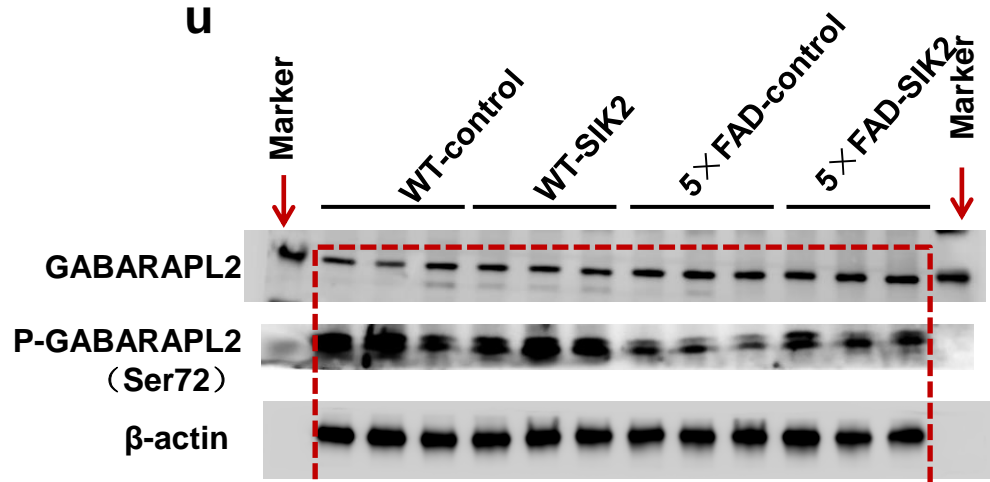

**w** N2a-APP IP: flag

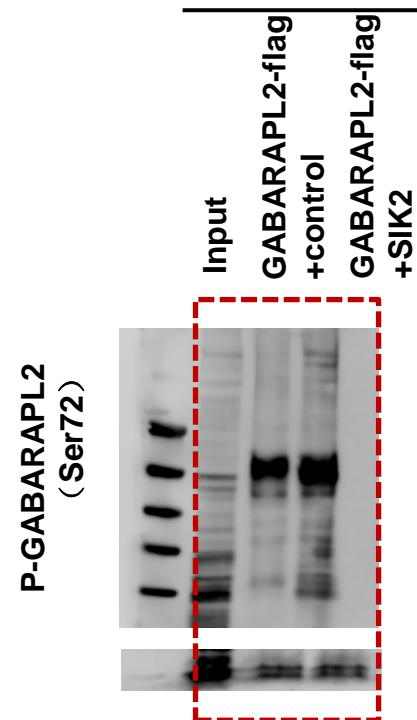

Fig6.

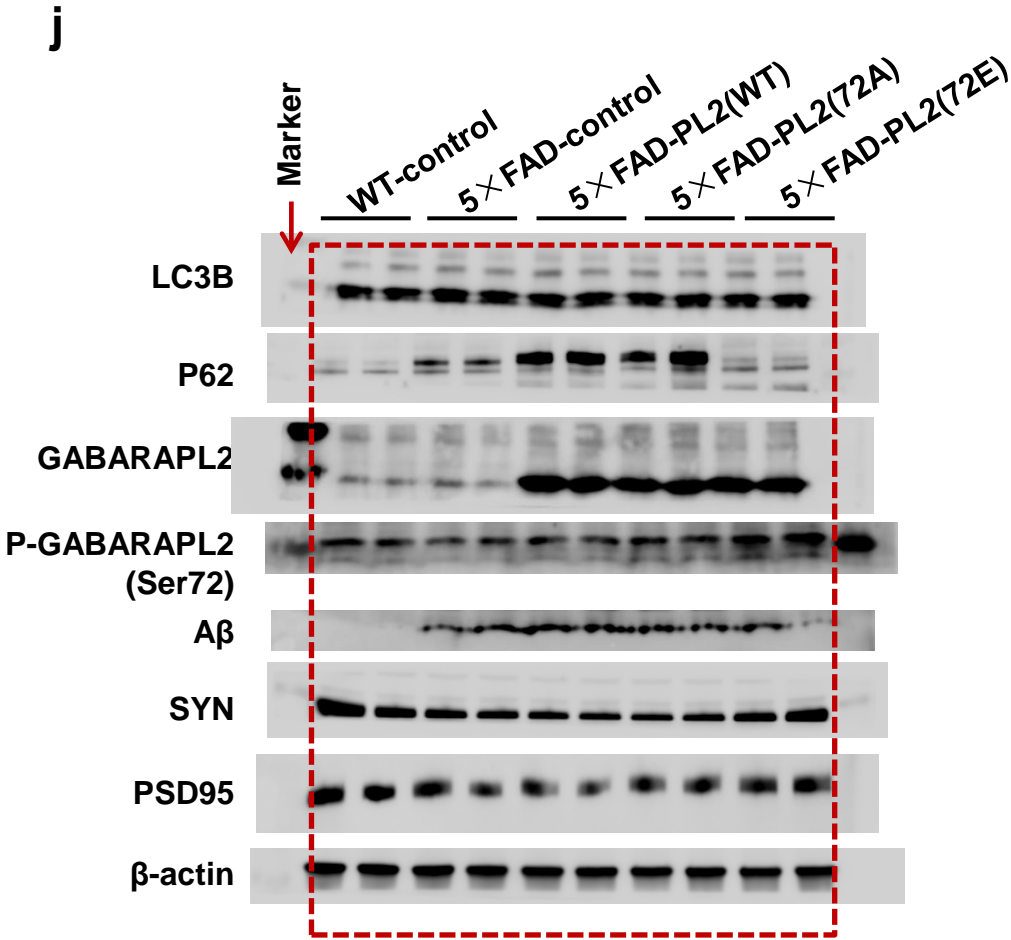

sFig1.

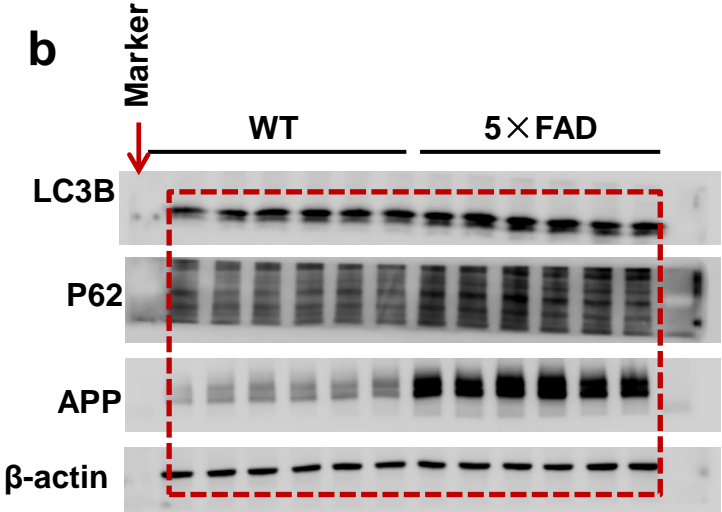

sFig2.

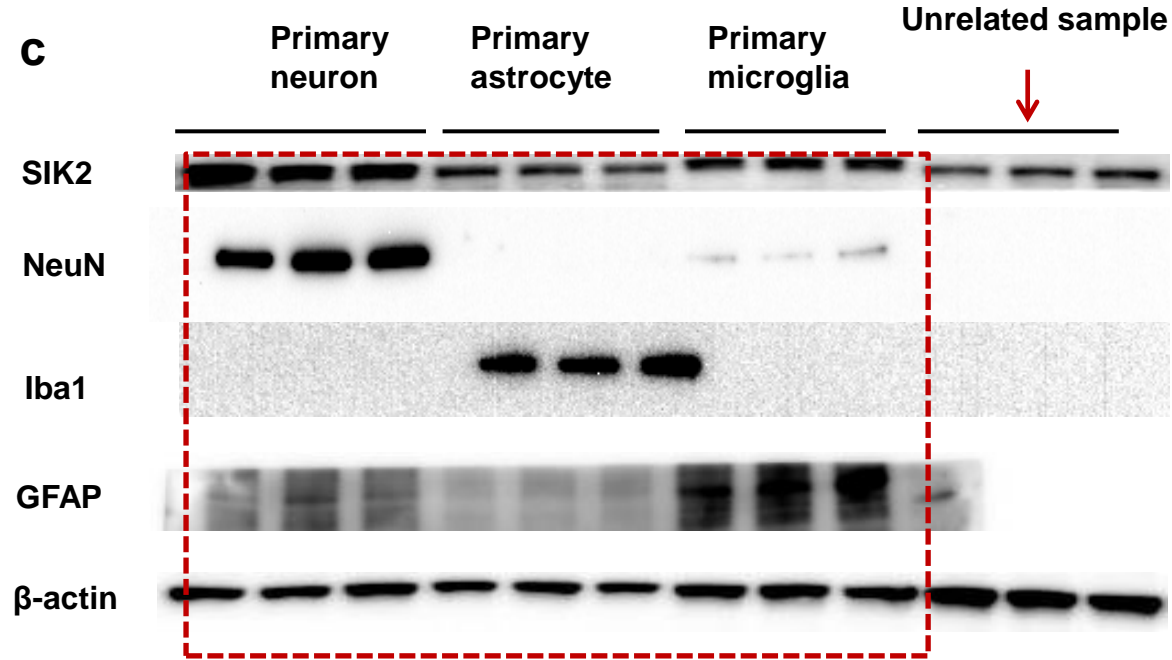

**sFig3.**

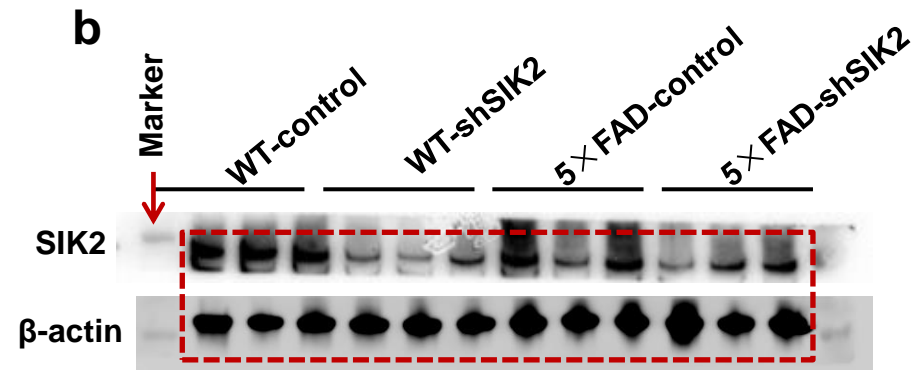

sFig4.

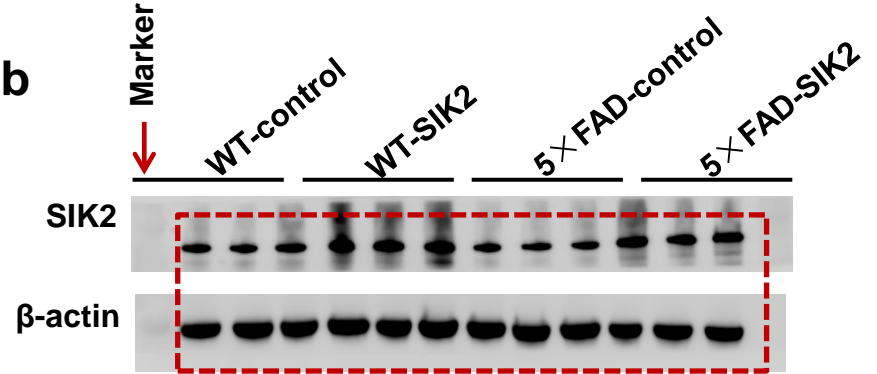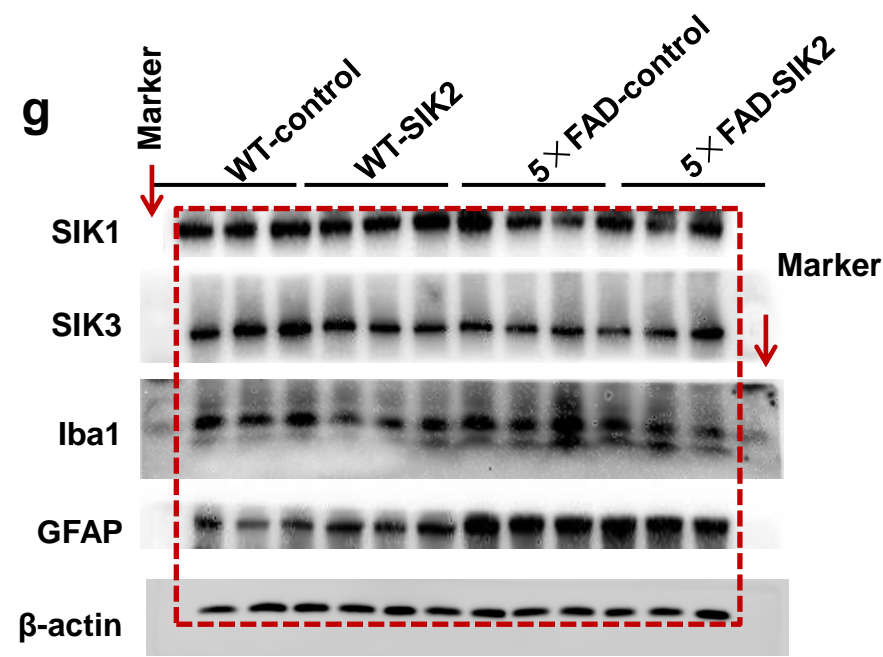

sFig5.

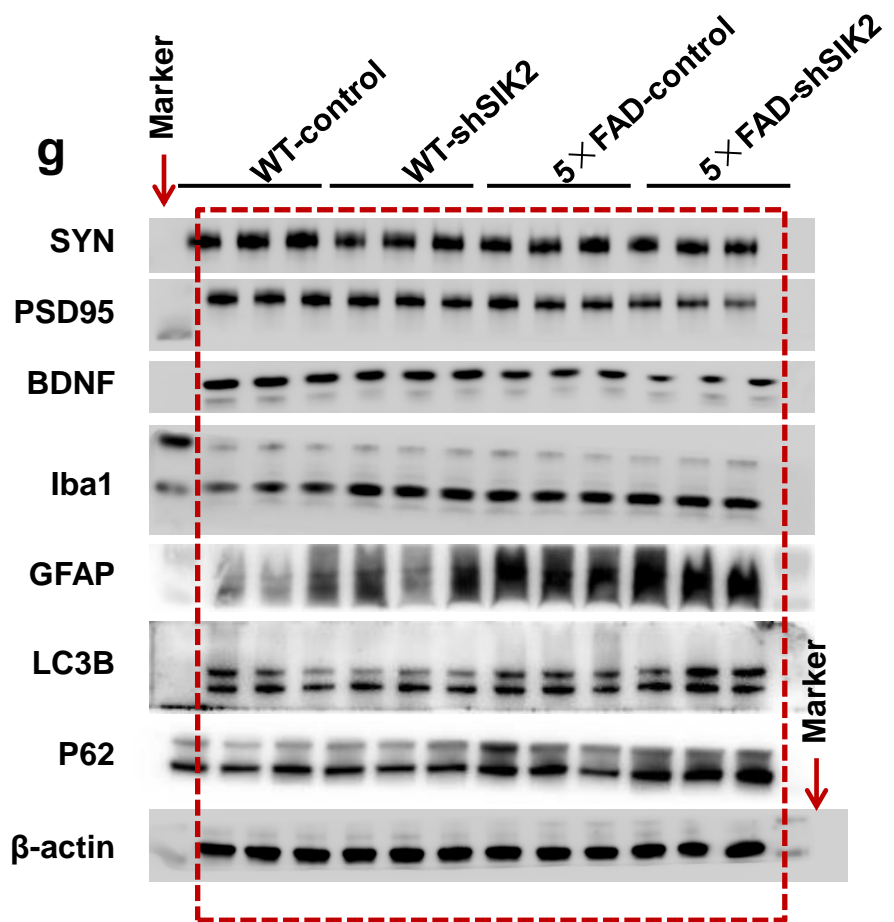

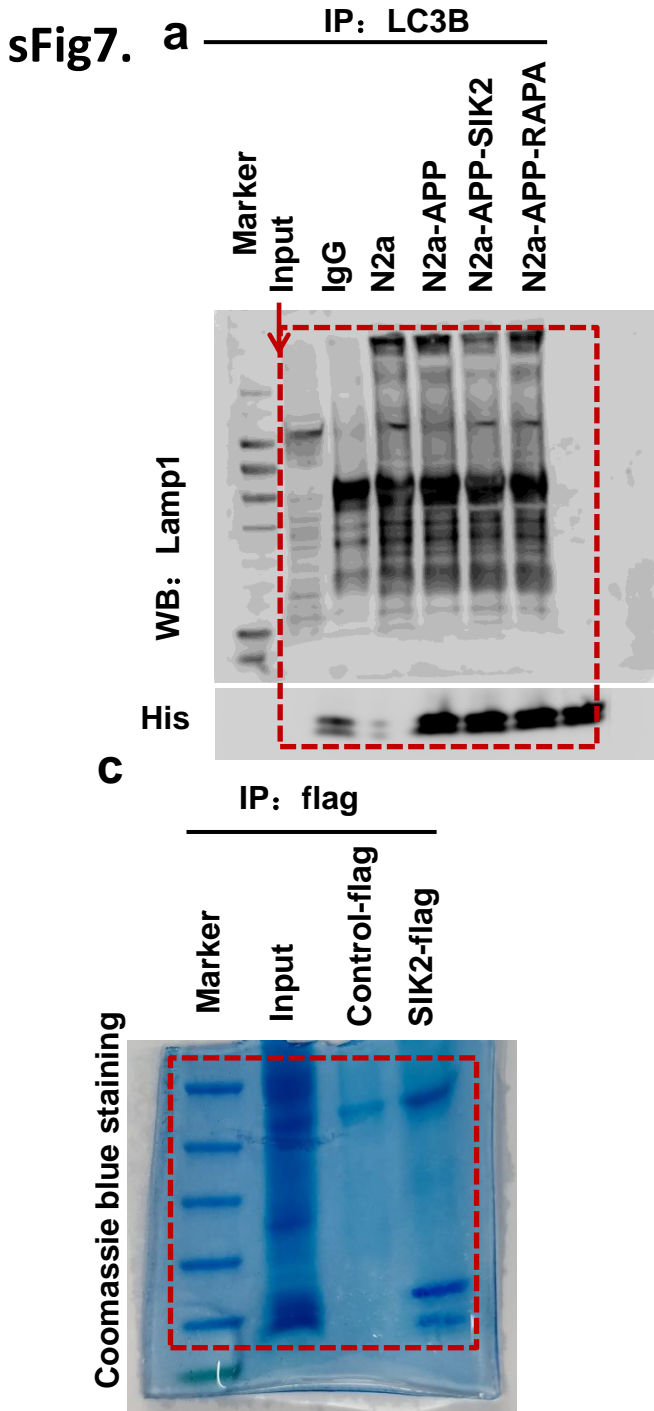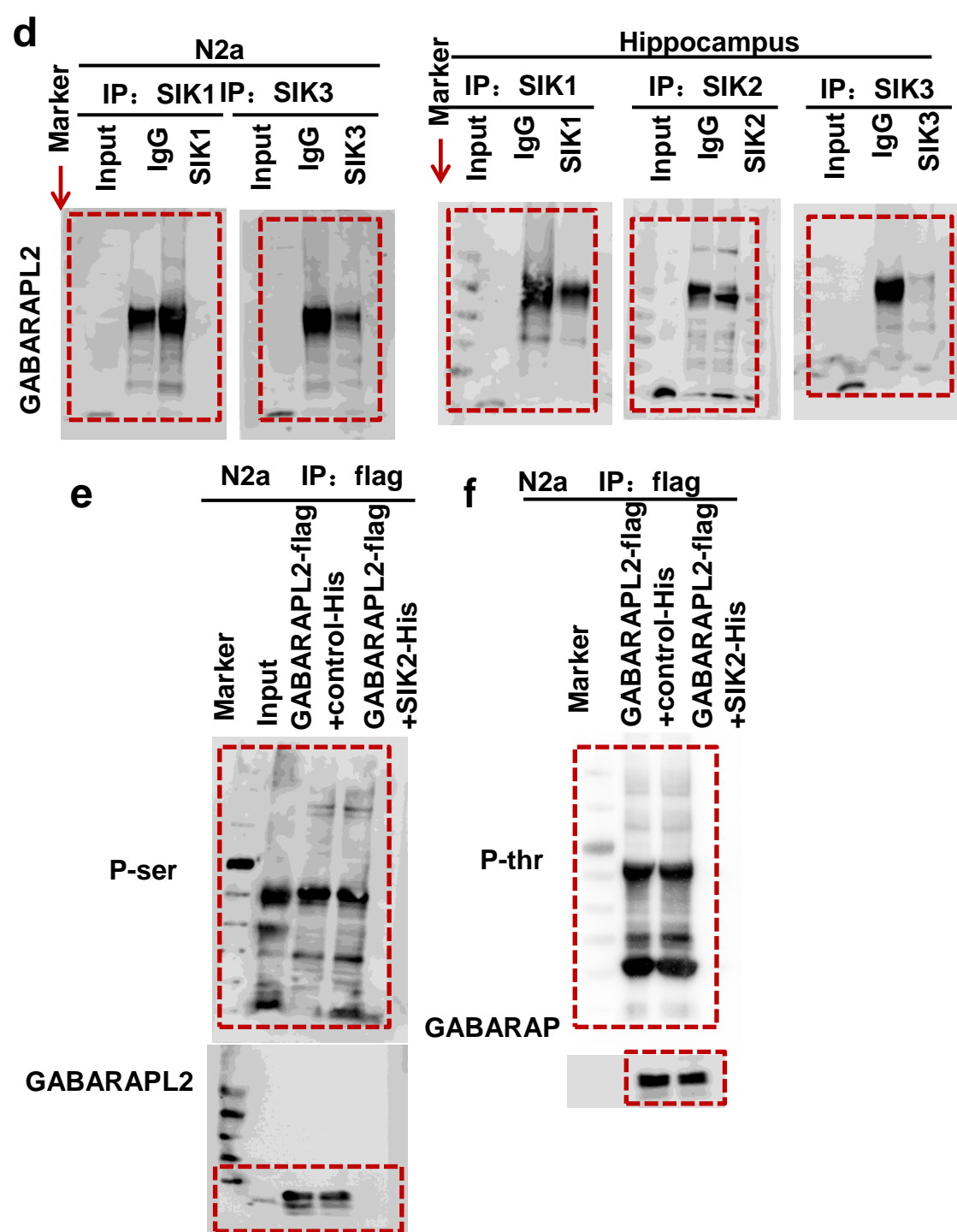

sFig7.

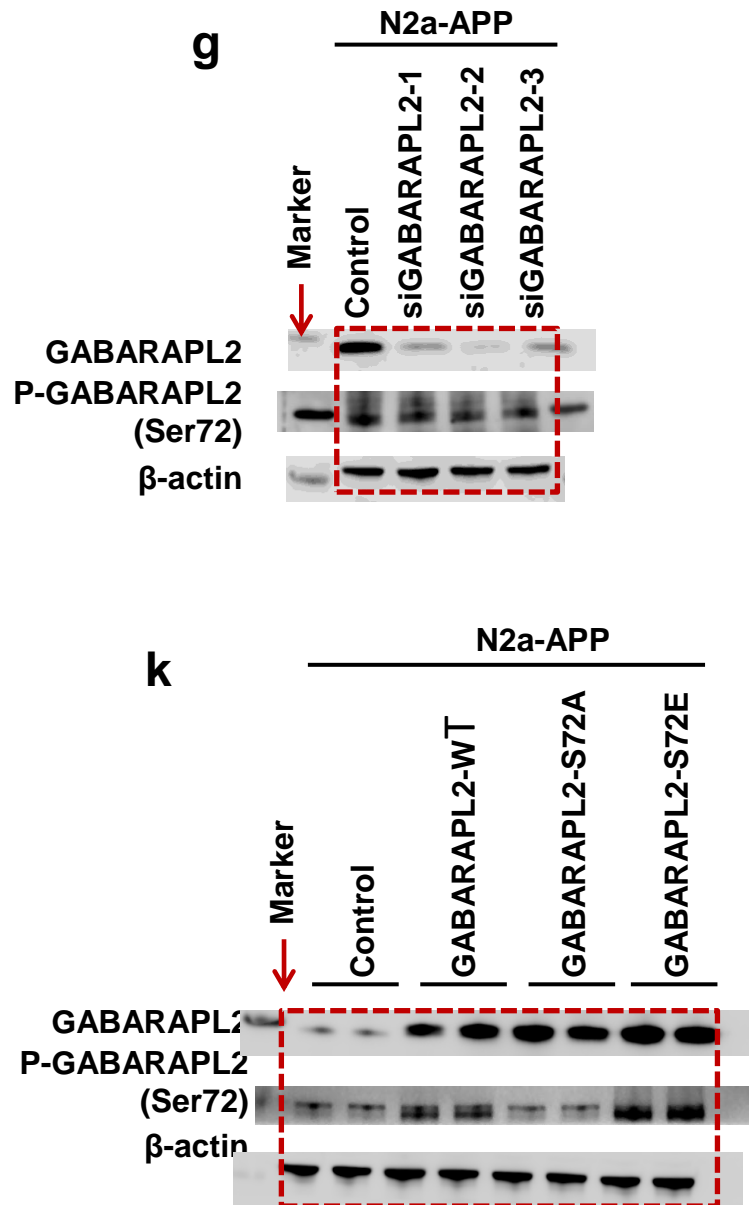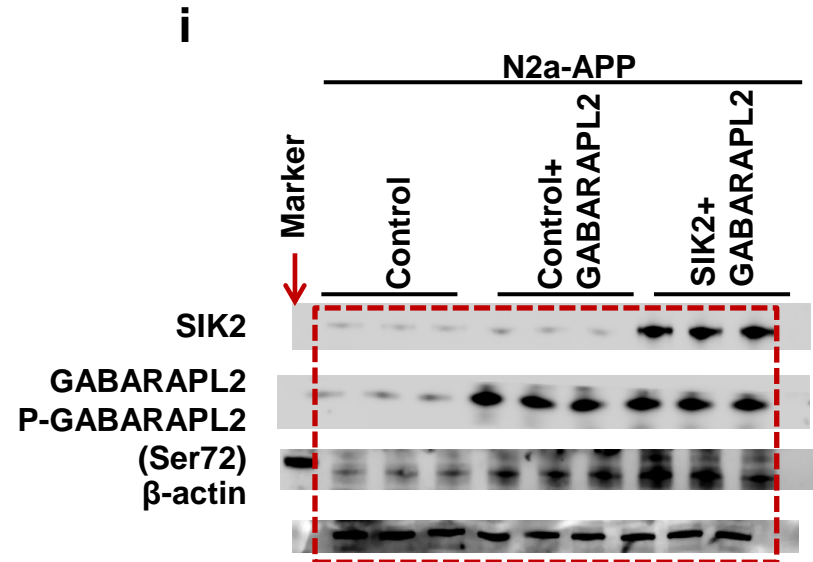

Supplement: Supplementary file 2 — Additional file 2. All original, full-length gel and blot images. [file 40035_2025_514_MOESM2_ESM.pdf]
